# Integrated immunovirological profiling validates plasma SARS-CoV-2 RNA as an early predictor of COVID-19 mortality

**DOI:** 10.1126/sciadv.abj5629

**Published:** 2021-11-26

**Authors:** Elsa Brunet-Ratnasingham, Sai Priya Anand, Pierre Gantner, Alina Dyachenko, Gaël Moquin-Beaudry, Nathalie Brassard, Guillaume Beaudoin-Bussières, Amélie Pagliuzza, Romain Gasser, Mehdi Benlarbi, Floriane Point, Jérémie Prévost, Annemarie Laumaea, Julia Niessl, Manon Nayrac, Gérémy Sannier, Catherine Orban, Marc Messier-Peet, Guillaume Butler-Laporte, David R. Morrison, Sirui Zhou, Tomoko Nakanishi, Marianne Boutin, Jade Descôteaux-Dinelle, Gabrielle Gendron-Lepage, Guillaume Goyette, Catherine Bourassa, Halima Medjahed, Laetitia Laurent, Rose-Marie Rébillard, Jonathan Richard, Mathieu Dubé, Rémi Fromentin, Nathalie Arbour, Alexandre Prat, Catherine Larochelle, Madeleine Durand, J. Brent Richards, Michaël Chassé, Martine Tétreault, Nicolas Chomont, Andrés Finzi, Daniel E. Kaufmann

**Affiliations:** 1 Research Centre of the Centre Hospitalier de l’Université de Montréal (CRCHUM), Montréal, QC, Canada.; 2 Département de Microbiologie, Infectiologie et Immunologie, Université de Montréal, Montréal, QC, Canada.; 3 Department of Microbiology and Immunology, McGill University, Montréal, QC, Canada.; 4 Department of Neuroscience, Université de Montréal, Montréal, QC, Canada.; 5 Centre hospitalier de l’Université de Montréal (CHUM), Montréal, QC, Canada.; 6 Lady Davis Institute, Jewish General Hospital, McGill University, Montréal, QC, Canada.; 7 Department of Epidemiology, Biostatistics and Occupational Health, McGill University, Montreal, QC, Canada.; 8 Department of Human Genetics, McGill University, Montreal, QC, Canada.; 9 Kyoto-McGill International Collaborative School in Genomic Medicine, Graduate School of Medicine, Kyoto University, Kyoto, Japan.; 10 Japan Society for the Promotion of Science, 5-3-1 Kojimachi, Chiyoda-ku, 102-0083 Tokyo, Japan.; 11 Department of Twin Research, King’s College London, London, UK.; 12 Département de Médecine, Université de Montréal, Montréal, QC, Canada.

## Abstract

Despite advances in COVID-19 management, identifying patients evolving toward death remains challenging. To identify early predictors of mortality within 60 days of symptom onset (DSO), we performed immunovirological assessments on plasma from 279 individuals. On samples collected at DSO11 in a discovery cohort, high severe acute respiratory syndrome coronavirus 2 (SARS-CoV-2) viral RNA (vRNA), low receptor binding domain–specific immunoglobulin G and antibody-dependent cellular cytotoxicity, and elevated cytokines and tissue injury markers were strongly associated with mortality, including in patients on mechanical ventilation. A three-variable model of vRNA, with predefined adjustment by age and sex, robustly identified patients with fatal outcome (adjusted hazard ratio for log-transformed vRNA = 3.5). This model remained robust in independent validation and confirmation cohorts. Since plasma vRNA’s predictive accuracy was maintained at earlier time points, its quantitation can help us understand disease heterogeneity and identify patients who may benefit from new therapies.

## Teaser

Statistical models of plasma immunovirological features validate SARS-CoV-2 vRNA as an early predictor of COVID-19 mortality.

## INTRODUCTION

Since the beginning of the pandemic, intense efforts have been deployed to define correlates of disease severity and to develop therapies targeting the virus or the pathogenesis of coronavirus disease 2019 (COVID-19). However, to date, only dexamethasone (*1*–*3*) and interleukin-6 (IL-6) blockers [tocilizumab (*4*) and sarilumab (*5*)] have convincingly shown to provide a survival benefit in randomized controlled trials. While other immune interventions may benefit some subgroups (*6*), there is currently no consensus on how to predict which critical cases are likely to resolve their infection and which are at a greater risk of fatality, in part due to the high heterogeneity of patients and the very dynamic changes in biological features (*2*).

Recent reports have identified features linked to severe COVID-19. One is high amounts of viral RNA (vRNA) in plasma, which has been associated with greater severity and worst outcome for other respiratory pathogens, such as severe acute respiratory syndrome coronavirus 1 (SARS-CoV-1) (*7*, *8*), respiratory syncytial virus (*9*), Middle East Respiratory Syndrome Coronavirus (MERS-CoV) (*10*), and the pandemic-causing strain of influenza A H5N1 (*11*). Plasma SARS-CoV-2 vRNA has also been linked to increased risk of severe COVID-19 and mortality (*12*–*17*).

Dysregulated immune responses are, at least in part, responsible for the exacerbated pathogenesis occurring in a minority of individuals with SARS-CoV-2 infection. Elevated cytokine levels were among the first reported markers associated to severe COVID-19 disease (*17*), although inconsistent sampling times sometimes led to weak associations with mortality (*18*). Narrowing the window of sampling early after symptom onset clarifies a plasma cytokine pattern (*19*) reminiscent of the cytokine release syndrome (*20*). Plasma profile around 10 days after symptom onset was highly differential for plasma cytokine profiles of critical versus moderate COVID-19 disease (*20*), and a number of cytokines have been associated with increased mortality (*21*).

Multiple studies support a central role for antibody responses in protective anti–SARS-CoV-2 immunity. The main viral target of antibody immunity is the trimeric Spike glycoprotein, which facilitates SARS-CoV-2 entry into host cells via interaction of its receptor binding domain (RBD) with angiotensin-converting enzyme 2 (ACE-2) (*22*, *23*). While most infected patients develop anti-Spike and anti-RBD antibodies (*24*, *25*), delayed anti-Spike immunoglobulin G (IgG) antibodies and decreased Fc effector capacity are associated with increased mortality (*26*). These reports highlight the complexity of the host’s immune response to SARS-CoV-2.

Despite the remarkable speed with which effective SARS-CoV-2 vaccines have been developed and deployed, partial population coverage and, potentially, emergence of resistant variants will lead to ongoing occurrence of infections. From a clinical perspective, it is therefore essential to identify a minimal set of early blood parameters that can be easily and rapidly measured to identify patients at high risk of mortality, while prioritizing parameters that may hint at specific categories of therapeutic interventions. However, the list of blood correlates of COVID-19 severity has tremendously expanded, making such prioritizing a major challenge. Given strong co–up-regulation between a number of plasma analytes [for example, plasma cytokines and chemokines; (*20*)], there is a need for streamlined analytical models with few virological and/or immunological parameters that provide complementary, rather than redundant, information to better stratify individual patient risk.

In this study, we simultaneously examined multiple parameters in plasma spanning three key aspects of COVID-19 pathogenesis early in disease course (11 ± 4 days after symptom onset, henceforth described as DSO11): SARS-CoV-2 vRNA, 26 cytokines and tissue injury markers, and 6 measures of SARS-CoV-2–specific antibody responses. We performed univariate and multivariate analyses to identify independent predictors of death. A minimal model combining vRNA, age, and sex was particularly robust, very reproducible in two additional cohorts, and remained predictive even when the samples were collected earlier in disease course.

## RESULTS

### Study design and patient characteristics

We investigated prospectively enrolled hospitalized COVID-19 individuals (*n* = 279) with symptomatic infection and with a positive SARS-CoV-2 nasopharyngeal swab (NSW) polymerase chain reaction (PCR), sampled longitudinally after enrollment. To allow for cross-sectional analysis of early plasma markers, we investigated patients for whom research blood samples were available at 11 (±4) days after symptom onset (DSO11) (*n* = 217). Our study population was split into a discovery cohort (*n* = 61) in a first hospital, a fully independent validation cohort (*n* = 87) in a second hospital (both of which were infected during the first wave), and a third confirmation cohort (*n* = 69) also collected in the first hospital, but mostly during the second and third waves (see fig. S1A). On the basis of disease severity at DSO11, patients were grouped as critical (requiring mechanical ventilation) versus noncritical (see participant characteristics, Table 1). The discovery cohort included 29 critical and 32 noncritical patients. Plasma profiles were compared to 50 asymptomatic uninfected donors as a control group [uninfected controls (UC)] of nondiseased state.

**Table 1.** Baseline characteristics of the participants and respiratory support at time of immunovirological profiling. Values displayed are medians, with IQR in parentheses for continuous variables, or percentages for categorical variables. Percentages are rounded to the nearest unit. “Noncritical illness” includes hospitalized patients with no oxygen support (no O_2_) (moderate disease) and oxygen support on nasal cannula (NC) only (severe, but noncritical disease). “Critical illness” includes hospitalized patients on mechanical ventilation, either positive pressure noninvasive ventilation (NIV), endotracheal intubation (ETI), and extracorporeal membrane oxygenation (ECMO). ICU admission and intubation are different in all cohorts between noncritical and critical due to selection bias (at *P* < 0.05) in any of the patient characteristic. For continuous variables, statistical test: Mann-Whitney *U* test, unpaired *t* test. For categorical variables, χ^2^ test.

	**Discovery cohort (*n* = 61)**			**Validation cohort (*n* = 87)**			**Confirmation cohort (*n* = 69)**		
**Variable**	**Noncritical**	**Critical**	**Entire cohort**	**Noncritical**	**Critical**	**Entire cohort**	**Noncritical**	**Critical**	**Entire cohort**
	**(*n*** ** = 32)**	**(*n*** ** = 29)**	**(*n*** ** = 61)**	**(*n*** ** = 68)**	**(*n*** ** = 19)**	**(*n*** ** = 87)**	**(*n*** = 42 or 24)*	**(*n*** = 27 or 13)*	**(*n*** = 69 or 37)*
Age	63 (49–80)	62 (51–68)	62 (49–73)^‡^	75 (57–88)	70 (55–73)	71 (56–84)^‡^	56 (49–71)^§^	70 (57–79)^§^	63 (51–75)
Sex									
Male	17 (53%)	20 (69%)	37 (61%)	33 (49%)	11 (58%)	44 (51%)	29 (69%)	16 (59%)	45 (65%)
Female	15 (47%)	9 (31%)	24 (39%)	35 (51%)	8 (42%)	43 (49%)	13 (31%)	11 (41%)	24 (34%)
Days since symptom onset	10 (8.5–13)	11 (10–12)	11 (9–12)	10 (8–12)	11 (9–12)	10 (9–12)	11 (10–12)	11 (10–13)	11 (10–13)
Days since hospital admission	5.5 (3–7)	5 (3–7)	5 (3–7)	4 (2–8)	5 (3–8)	5 (2–8)	5.5 (3–8.5)	5 (0–5)	5 (3–7)
Respiratory support									
No O_2_	20 (62%)	0 (0%)	20 (33%)^‡^	48 (71%)	0 (0%)	48 (55%)^‡^	23 (55%)	0 (0%)	23 (33%)
NC	12 (38%)	0 (0%)	12 (20%)^‡^	20 (29%)	0 (0%)	20 (23%)^‡^	19 (45%)	0 (0%)	19 (28%)
NIV	0 (0%)	7 (24%)	7 (12%)^‡^	0 (0%)	5 (26%)	5 (6%)^‡^	0 (0%)	15 (56%)	15 (22%)
ETI	0 (0%)	20 (69%)	20 (33%)^‡^	0 (0%)	14 (74%)	14 (16%)^‡^	0 (0%)	12 (44%)	12 (17%)
ECMO	0 (0%)	2 (7%)	2 (3%)^‡^	0 (0%)	0 (0%)	0 (0%)^‡^	0 (0%)	0 (0%)	0 (0%)
Total metabolic risk factors (0–4)	2 (1–3)	2 (1–3)	2 (1–3)						
None	3 (9%)	6 (21%)	9 (15%)						
One or more	29 (91%)	23 (79%)	52 (85%)						
Overweight, yes^†^	17 (53%)	21 (72%)	38 (62%)						
Hypertension, yes	20 (63%)	15 (52%)	35 (57%)	42 (62%)	13 (69%)	55 (63%)	9 (38%)	9 (69%)	18 (49%)
Dyslipidemia, yes	13 (41%)	11 (38%)	24 (39%)^‡^	11 (16%)	3 (16%)	14 (16%)^‡^	7 (29%)^§^	11 (85%)^§^	18 (49%)
Diabetes, yes	9 (28%)	10 (35%)	19 (31%)^||^	20 (29%)	9 (47%)	29 (33%)	8 (33%)^§^	11 (85%)^§^	19 (51%)^||^
Total chronic diseases (0–8)	0 (0–1)	0 (0–1)	0 (0–1)						
None	22 (69%)	17 (59%)	39 (64%)						
One or more	10 (31%)	12 (41%)	22 (36%)						
Chronic renal failure, yes	4 (13%)	6 (21%)	10 (16%)	9 (13%)	2 (11%)	11 (13%)	3 (13%)	3 (23%)	6 (16%)
Chronic heart failure, yes	2 (6%)	2 (7%)	4 (7%)	12 (18%)	2 (11%)	14 (16%)	2 (8%)	0 (0%)	2 (5%)
Chronic respiratory failure, yes	3 (9%)	5 (17%)	8 (13%)	6 (9%)	5 (26%)	11 (13%)	5 (21%)	0 (0%)	5 (14%)
Chronic liver failure, yes	0 (0%)	0 (0%)	0 (0%)	2 (3%)	0 (0%)	2 (2%)	0 (0%)	0 (0%)	0 (0%)
Organ transplant, yes	2 (6%)	2 (7%)	4 (7%)				n/a	n/a	n/a
Immunosuppression, yes	5 (16%)	4 (14%)	9 (15%)^‡^	2 (3%)	2 (11%)	4 (5%)^‡^	0 (0%)^§^	3 (25%)^§^	3 (8%)
Active cancer, yes	1 (3%)	3 (10%)	4 (7%)	9 (13%)	4 (21%)	13 (15%)	3 (13%)	0 (0%)	3 (8%)
HIV, yes	1 (3%)	1 (3%)	2 (3%)	1 (2%)	0 (0%)	1 (1%)	n/a	n/a	n/a
Total risk factors (metabolic/organ, 0–12)	2 (1–3)	3 (1–4)	2 (1–4)						
None	2 (6%)	6 (21%)	8 (13%)						
One or more	30 (94%)	23 (79%)	53 (87%)						
ICU admission, yes	3 (9%)^§^	27 (93%)^§^	30 (49%)^‡^	7 (10%)^§^	17 (90%)^§^	24 (28%)^‡^	2 (8%)^§^	12 (80%)^§^	14 (35%)
Intubation, yes	2 (6%)^§^	22 (76%)^§^	24 (39%)	7 (10%)^§^	17 (90%)^§^	24 (28%)	1 (4%)^§^	9 (75%)^§^	10 (29%)
Duration of intubation (days)	0 (0–0)^§^	20 (4–27)^§^	0 (0–18)				n/a	n/a	n/a
Duration of hospital stay (or in-hospital death)	10.5 (6–16)^§^	26 (14–44)^§^	16 (9–30)	14 (8–26.5)^§^	23 (19–48)^§^	18.5 (10–28)	10.5 (7.5–14.5)^§^	21 (13–34)^§^	12 (8–27)
**Outcome**									
Death up to 60 days									
Alive	30 (94%)^§^	18 (62%)^§^	48 (79%)	61 (90%)^§^	14 (74%)^§^	75 (86%)	42 (100%)^§^	16 (59%)^§^	58 (84%)
Dead	2 (6%)^§^	11 (38%)^§^	13 (21%)	7 (10%)^§^	5 (26%)^§^	12 (14%)	0 (0%)^§^	11 (41%)^§^	11 (16%)

*Only age, sex, and days since symptom onset variables have complete data in confirmation cohort; otherwise, the partial data are available for confirmation cohort.

†N_missing = 8 patients for discovery cohort.

‡Values are statistically different between discovery and validation cohorts.

§Values are statistically different between critical and noncritical groups.

||Values are statistically different between discovery and confirmation cohorts.

We clinically followed participants for at least 60 days after symptom onset (DSO60). The primary outcome, death by DSO60, occurred in 13 patients, with close to half fatalities occurring between DSO30 and DSO60 (fig. S1B) and mostly in the critical group (fig. S1C).

We performed a slightly reduced immunovirological assessment in the validation cohort, where 19 cases were critical and 12 deaths occurred before DSO60, and a focused assessment of the confirmation cohort (with 27 critical cases and 11 fatalities) (Table 1). Because of hospital referral coordination, the validation cohort was of older age than the discovery one, but with less severe respiratory compromise (Table 1). Other basic demographics and prevalent risk factors were consistent with published studies (*27*) and, overall, showed minor differences between all cohorts. These features did not significantly differ between the critical versus noncritical groups, except for higher rates of admission to intensive care unit (ICU) and intubation and duration of hospital stay in critical patients (Table 1), in line with group definition. Last, for sensitivity longitudinal measurements of the selected statistical models through different DSO points, we complemented these three cohorts with 62 patients who were sampled very early in disease course (before the DSO11 ± 4 days time bracket).

### Plasma viral load in early disease is strongly associated with COVID-19 mortality

**Fig. 1.**
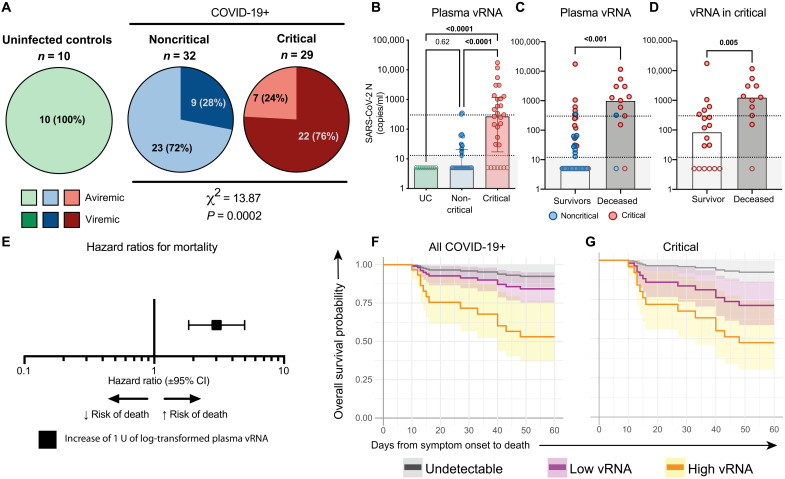
High quantity of SARS-CoV-2 RNA in plasma at DSO11 is associated with increased risk of mortality. (**A**) Pie charts representing the fractions of assessed samples that had undetectable (aviremic, light shades, <13 copies/ml) or detectable SARS-CoV-2 vRNA (dark shades, ≥13 copies/ml). Numbers in parts refer to the number (and percentages) of patients within each cohort. Noncritical and critical subgroups compared by χ^2^ test. (**B**) Quantities of SARS-CoV-2 N copies detected per milliliter of plasma in each cohort. Dotted line is the limit of detection (13 copies/ml). Empty shapes have undetectable vRNA (arbitrarily set at 5 copies/ml for representation). (**C** and **D**) Amounts of SARS-CoV-2 N copies detected per milliliter of plasma in patients who survived (white column) or died (gray column) by DSO60 for (C) total cohort or (D) critical subgroup only. Red circles represent critical patients, and blue circles are noncritical. (**E**) HR with 95% CI calculated using Cox regression for an increase of 1 U of log_10_-transformed vRNA (copies/ml). (**F** and **G**) Modelization of the predicted survival curves of patients with high [orange; upper interquartile range (IQR)], low (purple; lower IQR), or undetectable (gray) plasma vRNA in (F) all patients with COVID-19 or (G) critical cases only. (B) Kruskal-Wallis with Dunn’s multiple comparisons test. (C) Mann-Whitney test. *n* = 61 COVID-19 subjects (13 mortalities) or 29 critical COVID-19 cases (11 mortalities) and 10 UC. IQR: calculated among detectable vRNA quantities only.

As SARS-CoV-2 vRNA in plasma has been previously linked to mortality, we quantified it in the discovery cohort. We designed an ultrasensitive quantitative real-time PCR (qRT-PCR) targeting the N sequence of its genome with a detection limit of 13 copies/ml. The assay was highly specific, with no vRNA detected in UC (Fig. 1A). At DSO11, we detected plasma SARS-CoV-2 vRNA in a significantly greater fraction of critical than noncritical patients (Fig. 1A). These results suggest that systemic SARS-CoV-2 viremia is a signature of infection severity and/or itself plays a role in disease complications.

We next hypothesized that the amount of viral products, rather than their mere presence, was associated with severe pathogenesis. SARS-CoV-2 vRNA levels were higher in critical than noncritical cases (Fig. 1B). This difference held when comparing samples with detectable levels only (*P* = 0.002, Mann-Whitney test). Most patients who died had high vRNA compared to survivors (Fig. 1C), even when the analysis was restricted to critical cases (Fig. 1D). In univariate Cox regression analysis (Table 2), we found that an increase of 1 U in log-transformed plasma vRNA led to a threefold increase in mortality risk {hazard ratio [HR] = 3.1 [95% confidence interval (CI): 1.9 to 5.1], *P* < 0.0001 for all COVID-19 [Fig. 1E] and 2.5 [95% CI: 1.4 to 4.7; *P* = 0.004] for critical [Table 2]}. The estimated survival proportions for undetectable (<13 copies/ml), low, or high plasma vRNA were extracted from Cox models (see Materials and Methods for details) (*28*). High plasma vRNA was associated with a greater risk of death, whereas there was a substantial overlap between the subgroups with low or undetectable plasma vRNA (Fig. 1F). A similar trend was observed in the critical group (Fig. 1G). Therefore, plasma SARS-CoV-2 vRNA load is not only a correlate of contemporaneous respiratory compromise early in disease course but also associated with mortality, including in the critical group.

**Table 2.** Univariate Cox proportional hazard regression of single variables measured in COVID-19 patient plasma at DSO11. RLU, relative light units, normalized to internal control (CR3022) (see Materials and Methods for details); MFI, mean fluorescence intensity; ID_50_, neutralization half-maximal (50%) inhibitory dilution.

	**Discovery cohort**			
**Variable**	**All COVID-19–positive at DSO11 (*n* = 61)**		**Critical COVID-19–positive subset at DSO11 (*n* = 29)**	
	**HR (95% CI)**	***P***	**HR (95% CI)**	***P***
	**1 U**		**1 U**	
**Viral load**				
vRNA (copies/ml of plasma)*	**3.1 (1.9–5.1)**	**<0.001**	**2.5 (1.4–4.7)**	**0.004**
**Serology**				
RBD-specific IgG (RLU)*	**0.3 (0.1–0.8)**	**0.011**	**0.3 (0.1–0.7)**	**0.005**
RBD-specific IgM (RLU)*	0.5 (0.2–1.4)	0.186	0.4 (0.1–1.3)	0.144
RBD-specific IgA (RLU)*	**0.4 (0.2–0.98)**	**0.045**	**0.3 (0.1–0.8)**	**0.014**
Spike Ig (MFI)*	**0.6 (0.5–0.9)**	**0.006**	**0.5 (0.3–0.8)**	**0.002**
Neutralization (ID_50_)*	0.8 (0.6–1.1)	0.172	**0.7 (0.5–0.9)**	**0.020**
ADCC (%)^†^	**0.7 (0.5–0.9)**	**0.006**	**0.1 (0.5–0.9)**	**0.004**
**Cytokines**				
Angiopoietin-2*	**14.5 (3.4–62.1)**	**0.001**	**6.7 (1.4–33.0)**	**0.018**
CCL2/JE/MCP-1*	**5.6 (1.7–18.4)**	**0.005**	2.8 (0.8–10.2)	0.115
CCL20/MIP-3 alpha*	**2.9 (1.2–6.8)**	**0.016**	1.4 (0.5–4.0)	0.578
CCL7/MCP-3/MARC*	4.0 (1.0–15.6)	0.050	5.2 (0.9–30.7)	0.068
CD40 Ligand/TNFSF5*	**5.6 (1.0–30.8)**	**0.049**	6.7 (0.8–55.7)	0.080
CXCL10/IP-10/CRG-2*	16.7 (0.7–423.5)	0.088	5.5 (0.2–161.2)	0.323
CXCL13/BLC/BCA-1*	**6.7 (2.6–17.2)**	**<0.001**	**4.3 (1.6–11.7)**	**0.005**
IL-8/CXCL8*	**5.6 (1.7–18.6)**	**0.005**	**3.8 (1.0–14.3)**	**0.048**
CXCL9/MIG*	2.2 (0.8–6.4)	0.133	1.2 (0.5–2.9)	0.621
D-dimer*	5.0 (0.5–49.9)	0.174	0.4 (0.02–8.5)	0.548
G-CSF*	**3.3 (1.1–10.2)**	**0.034**	**3.5 (1.1–10.8)**	**0.032**
GM-CSF*	**9.4 (1.7–50.7)**	**0.009**	**7.3 (1.02–51.4)**	**0.047**
IFNα*	2.4 (0.9–6.5)	0.087	2.4 (0.8–7.0)	0.114
IL-1ra/IL-1F3*	**8.0 (1.9–33.7)**	**0.004**	2.8 (0.6–14.4)	0.214
IL-23*	**13.7 (2.2–85.6)**	**0.005**	**7.2 (1.1–46.3)**	**0.038**
IL-6*	**2.6 (1.4–5.0)**	**0.003**	1.5 (0.7–3.3)	0.315
SP-D*	**5.2 (1.0–26.3)**	**0.047**	2.2 (0.3–15.7)	0.433
TNFα*	**16.5 (2.7–102.5)**	**0.003**	6.6 (0.9–50.9)	0.069
RAGE/AGER*	**7.9 (2.3–27.9)**	**0.001**	**4.4 (1.01–18.9)**	**0.049**
CytoScore^‡^	**2.6 (1.6–4.2)**	**<0.001**	**1.9 (1.1–3.4)**	**0.0335**

*Variables are log_10_-transformed. HR shown is for an increase of 1 U of log_10_-transformed variable.

†HR for increase of 10 U.

‡Refer to Materials and Methods for details.

### Markers of immune hyperactivation and tissue damage discriminate disease trajectories

**Fig. 2.**
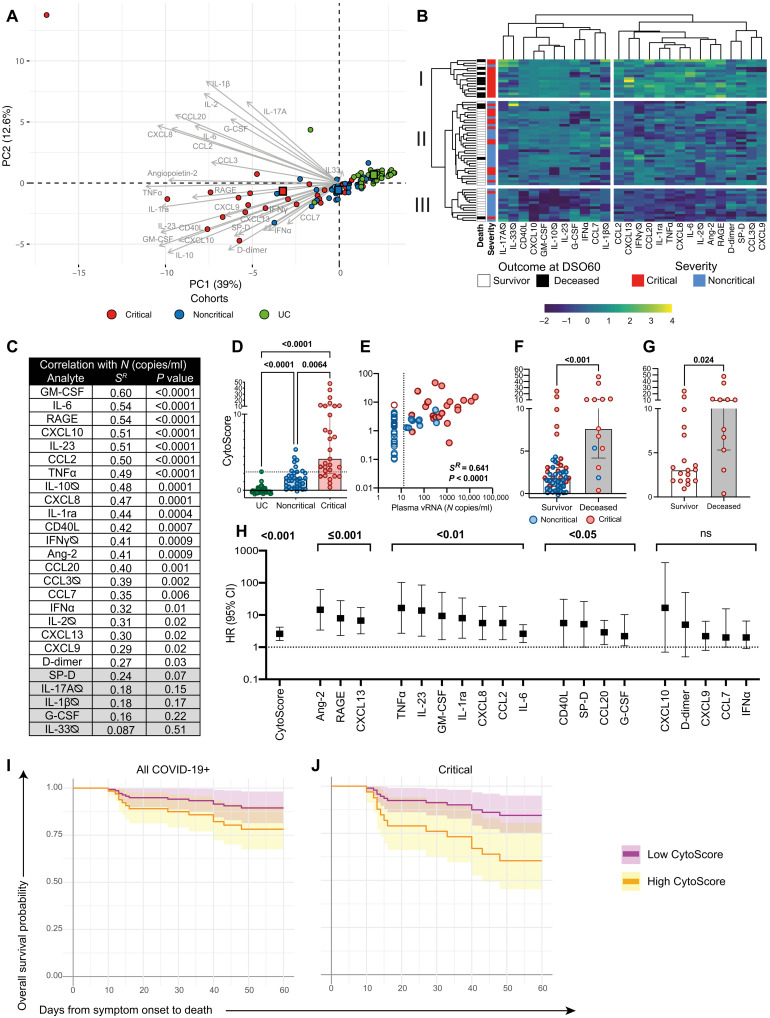
High cytokine titers in plasma at DSO11 discriminates critical disease and is associated with increased risk of mortality. (**A**) PCA representation of critical and noncritical patients (at DSO11), and UC (at baseline), on the basis of the 26 plasma analytes. Color-coded squares represent the mean PC (principal component) coordinates for each group. Length of arrow indicates the contribution of analytes to PCs. Numbers in parentheses along axes are the percentage of variance that PC accounts for. (**B**) Heatmap analysis of log-transformed concentrations of all 26 plasma analytes (yellow: high relative expression; blue: low relative expression), with unsupervised hierarchical clustering of the analytes (top dendrogram) or of patients (left dendrogram). The leftmost column represents outcome at DS60 (white: survival; black: deceased). The following column is the severity of the patient at DSO11. (**C**) Table showing the Spearman *R* values and corresponding *P* values of correlation of each plasma analyte with plasma vRNA. Values shaded in gray are nonsignificant. (**D**) Comparison of CytoScore of each cohort (see Materials and Methods for details on CytoScore). (**E**) Correlation between plasma vRNA and CytoScore. Empty shapes are aviremics (<13 copies of SARS-CoV-2 N copies/ml of plasma). (**F** and **G**) CytoScore of patients who survived (white column) or deceased (gray column) by DSO60 for (F) all patients with COVID-19 or (G) critical subgroup only. (**H**) HR with 95% CI calculated using Cox regression for a 1-U increase in the log_10_-transformed concentration of each plasma analyte with robust detection (see Materials and Methods for details) and CytoScore. ns: not significant. (**I** and **J**) Modelization of the predicted survival curves of patients with high (orange; upper IQR) or low (purple; lower IQR) CytoScore in (I) all patients with COVID-19 or (J) critical subgroup only. (C and E) Spearman correlations. (D) Kruskal-Wallis with Dunn’s multiple comparisons test. (F) Mann-Whitney test. For (A), (B), and (D) to (F), color-coded dots represent severity of the patient at DSO11 (red: critical; blue: noncritical) or UC cohort (green). (B and D) Cytokines with titles annotated by ∅ are poorly detected (see Materials and Methods for details). *n* = 61 COVID-19 subjects (13 mortalities) or 29 critical COVID-19 cases (11 mortalities) and 43 UC. IQR: calculated within the CytoScores of the COVID-19 discovery cohort.

As early elevation of a number of cytokines and chemokines was also associated with adverse COVID-19 outcome (*19*, *20*, *29*), we used multiplex bead array to determine plasma levels of the 26 proteins associated with adaptive and/or innate immune responses, chemotaxis, or tissue insult related to severe acute respiratory distress syndrome (ARDS; see table S1 for analyte list). Principal components analysis (PCA) revealed that the plasma profile largely delineates UC from patients with COVID-19 and highlighted higher cytokine levels and greater heterogeneity in the critical group compared to the noncritical group (Fig. 2A). The outlier critical case at the upper left corner of the PCA was on extracorporeal membrane oxygenation (ECMO) at the time of sampling, a procedure known to affect plasma profile (*30*). Unsupervised hierarchical clustering solely based on the 26 measured plasma proteins parsed apart three patient clusters: (i) mostly critical, (ii) mixed, and (iii) mostly noncritical cases (Fig. 2B).

We next compared the levels of each analyte between groups (fig. S2, A to D). Several followed a stepwise increase, where noncritical cases had greater cytokine concentrations than UC, and critical cases had the greatest amounts (fig. S2A). These included pro-inflammatory cytokines and chemokines IL-6, GM-CSF (granulocyte-macrophage colony-stimulating factor), TNFα (tumor necrosis factor–α), CCL2, and CXCL8. Some of the markers of tissue insult [RAGE (receptor for advanced glycation end products) and Angiopoietin-2] (*31*) also increased with disease severity, likely reflecting the extent of lung and vascular damage. CXCL9, CD40L, IFNα (interferon-α), and surfactant pulmonary protein D (SP-D) were significantly greater only in the critical cases of COVID-19 compared to UC (fig. S2B), while a few markers did not differ between all three groups (fig. S2C). Some analytes were significantly elevated in COVID-19 groups but did not differ between the critical and noncritical groups, such as CXCL10 (IP10), CXCL13, and D-dimer (fig. S2D). Together, the plasma profile reveals overall higher quantities of cytokines in the plasma of patients with COVID-19 compared to UC, and select analytes are specifically associated with greater disease severity.

We reasoned that these 26 analytes may be differentially linked to the amount of vRNA in plasma. We examined the correlations between individual plasma analytes (fig. S2E), as well as their association with vRNA (Fig. 2C). Many analytes were co-upregulated, and several of them also positively correlated with vRNA levels. These latter correlations were particularly robust for cytokines implicated in innate immune responses such as IL-6 (fig. S2F) and GM-CSF; the marker of lung damage RAGE (fig. S2G); and inflammatory chemokines CXCL8, CXCL10, and CCL2, suggesting a shared trigger or overlap in pathways.

To capture by a single parameter the overall magnitude of the difference in cytokine titers between patients with COVID-19 and UC, we created a “CytoScore” from the linear combination of all 26 analytes (see Materials and Methods for details). It followed a gradual difference, where the noncritical group had lower CytoScores than the critical group, and UC had the lowest scores (Fig. 2D). The CytoScore correlated positively with vRNA (Fig. 2E) and can have value in reducing dimensionality of plasma analyte profiling.

As patients who died within DSO60 showed a greater CytoScore than survivors (Fig. 2F), even when restricted to critical cases (Fig. 2G), we applied Cox regression analyses to examine the association between the cytokines and mortality over time. We focused on analytes whose concentrations are in the range of robust quantitation by the assay (19 of 26; see Materials and Methods for details). For each, we calculated the HR associated with a 1-U increase in log-transformed concentration (Fig. 2H). In addition to the CytoScore, several individual analytes were significantly associated with increased fatality risk, with Angiopoietin-2, RAGE, and CXCL13 showing the highest significance (*P* ≤ 0.001). Furthermore, patients with high CytoScore at DSO11 showed a significantly lower rate of predicted survival at DSO60 than the low CytoScore population, both in the entire discovery cohort (Fig. 2I) and in the critical group (Fig. 2J). Therefore, overall cytokine levels as well as individual cytokines and markers of tissue damage measured at DSO11 are (i) in majority correlated with plasma vRNA and (ii) associated with increased risk of mortality among patients with COVID-19.

### Low SARS-CoV-2–specific IgG and limited ADCC associated with COVID-19 mortality

**Fig. 3.**
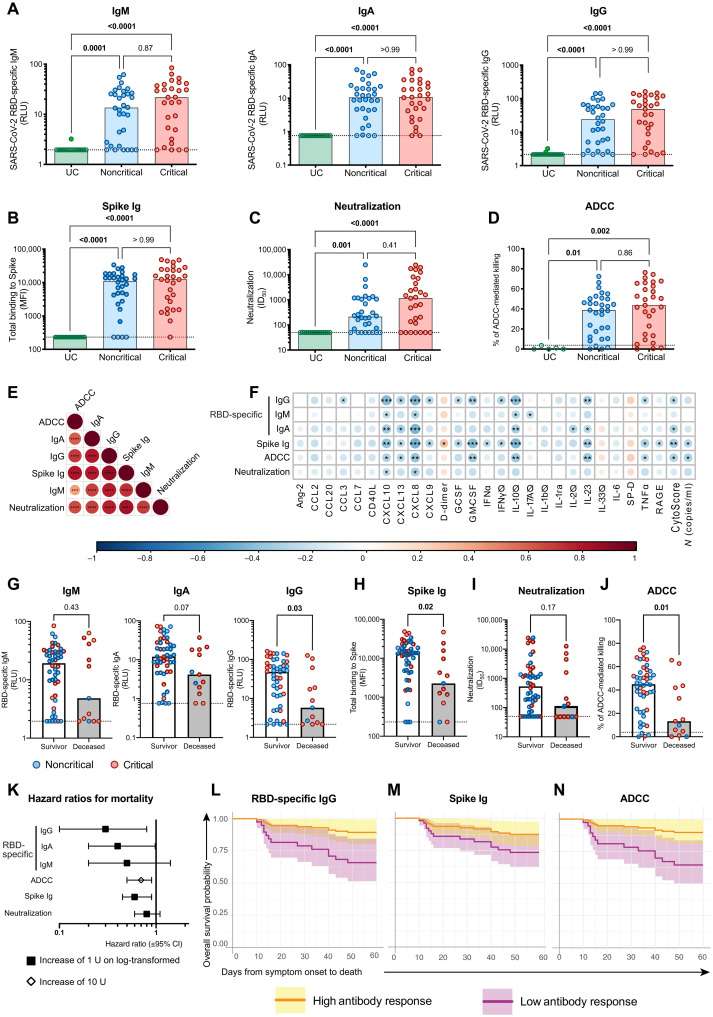
Limited IgG responses against SARS-CoV-2 Spike at DSO11 are associated with mortality. (**A**) ELISA-based relative quantification of SARS-CoV-2 RBD-specific antibodies’ isotypes IgM (left), IgA (middle), or IgG (right) in relative light units (RLU) normalized to an internal control (CR3022). (**B** to **D**) Comparison of functional properties of the plasma of all three groups, namely, (B) plasma capacity to recognize the SARS-CoV-2 full Spike (Spike Ig) using a flow cytometry–based assay [median fluorescence intensity (MFI)], (C) plasma neutralization activity [unit: half of maximal inhibitory plasma dilution (ID_50_)], and (D) plasma ADCC activity (unit: % of ADCC-mediated killing). (**E** and **F**) Correlation matrices with colors representing the Spearman *R* value (blue: negative association −1; red: positive association 1) and *P* values indicated as * in the circles, (E) between all serology measurements or (F) of serology measurements versus plasma vRNA and plasma analytes. (**G** to **J**) Comparison of serology measurements in patients who survived (white column) or deceased (gray column) by DSO60 for (G) RBD-specific IgM (left), IgA (middle), or IgG (right) or (H) full Spike binding, (I) neutralization, or (J) ADCC. (**K**) Hazard ratio with 95% CI calculated using Cox regression for an increase of 1 U of log_10_-transformed (square) or 10 U (diamond) of serology measurements. (**L** to **N**) Modelization of the predicted survival curves of patients with high (orange; upper IQR) or low (purple; lower IQR) (L) RBD-specific IgG, (M) Spike Ig, or (N) ADCC activity in all patients with COVID-19. (A to D) Kruskal-Wallis with Dunn’s multiple comparisons test. (E and F) Spearman *R* correlation. (F) Cytokines with titles annotated by ∅ are poorly detected. (G to J) Mann-Whitney test. For (G) to (J), color-coded dots represent severity of the patient at DSO11 (red: critical; blue: noncritical), and the dotted line represents the limit of detection. (E, F, and L) *P < 0.05; **P < 0.01; ***P < 0.001. *n* = 61 COVID-19 subjects (13 mortalities) or 29 critical COVID-19 cases (11 mortalities) and 43 UC. IQR: calculated within the COVID-19 discovery cohort.

As SARS-CoV-2 antibody responses likely play a critical role in protective immunity against SARS-CoV-2 (*26*, *32*), we measured plasma SARS-CoV-2–specific antibody responses at DSO11. Enzyme-linked immunosorbent assay (ELISA)–based quantification using the SARS-CoV-2 RBD protein and isotype-specific secondary antibodies (*24*, *33*) revealed a broad range in relative quantities of RBD-specific IgM, IgA, or IgG in the noncritical and critical groups at DSO11. They did not differ between groups and were not detected in UC (Fig. 3A). These observations were corroborated by a flow cytometry–based assay measuring plasma binding to full-length Spike protein (Spike Ig) on cell surface (Fig. 3B), which similarly showed no notable difference between the two COVID-19 groups.

We next assessed the SARS-CoV-2 Spike-specific antibody response for two key antiviral functions: neutralization (Fig. 3C) and antibody-dependent cellular cytotoxicity (ADCC; Fig. 3D). Here, again, the data showed high variability and no significant differences between the critical and noncritical groups for both readouts. All serology measurements were interrelated (Fig. 3E). In contrast, the serology measurements were inversely correlated with plasma vRNA and most cytokines (Fig. 3F).

To assess potential consequences of defective antibody responses at this early time point, we compared SARS-CoV-2–specific antibody responses between survivors and nonsurvivors. For RBD-specific isotypes (Fig. 3G), only IgG amounts were significantly increased in survivors, although there was a similar trend for IgA as well. Spike Ig levels were also higher in survivors (Fig. 3H). We observed contrasting patterns with regard to functional humoral responses: While neutralization capacity was similar for both outcomes (Fig. 3I), ADCC capacity was superior in survivors (Fig. 3J). HR reflected the same observations, where higher ADCC, RBD-specific IgG, and Spike Ig were associated with increased survival (Fig. 3K). We further modeled this by comparing the survival curves at DSO60 of patients with low or high RBD-specific IgG amounts (Fig. 3L), Spike Ig (Fig. 3M), or ADCC (Fig. 3N) at DSO11 and saw that participants with low responses for these three measurements showed an increased fatality risk. These observations were maintained when the analysis was restricted to the critical group (fig. S3, A to C). Together, these results highlight that impairment of some SARS-CoV-2–specific antibody responses may contribute to mortality.

### Multivariate Cox reveal plasma vRNA as pivotally associated with COVID-19 mortality

As all categories of immunovirological parameters showed some perturbations that predicted fatality, we examined whether these alterations provided redundant information in terms of mortality risk, or whether their combined analysis would improve associations with fatal outcome. Within immunovirological categories, we retained only variables significant in univariate Cox analysis (*P* < 0.05; see Table 2), and among those, a global multivariate model was used to select top variables (see Materials and Methods for details). To evaluate predictive accuracy of the resulting variables in multivariate models, we calculated time-dependent receiver operator characteristic (ROC) curves at DSO60 (principles are illustrated in fig. S4A). The area under the curve (AUC), a measure of prediction accuracy, was examined at all distinct event times by plotting the AUC over time (principles are illustrated in fig. S5A; see Materials and Methods for details). All final time-dependent Cox models were reassessed in the validation cohort to validate the accuracy of our findings.

**Fig. 4.**
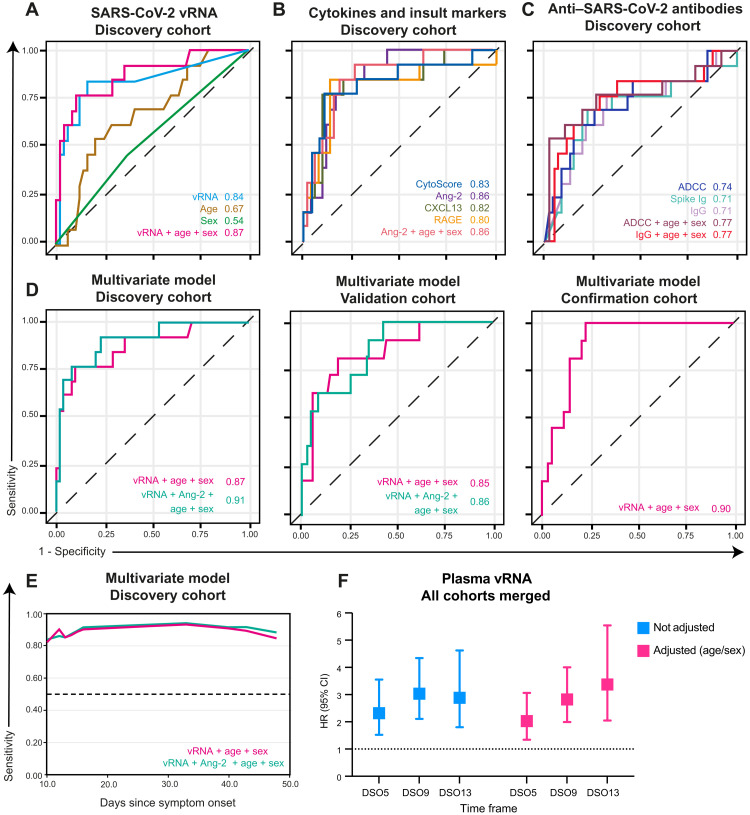
Time-dependent ROC curves reveal plasma vRNA as reproducibly associated with mortality in the discovery, validation, and confirmation cohorts. (**A** to **C**) Time-dependent ROC curves measured within the discovery cohort for (A) plasma vRNA, age, and sex; (B) cytokines and tissue insult markers; or (C) anti–SARS-CoV-2 antibody responses. (**D**) Time-dependent ROC curves of top multivariate models selected by Bayesian information criterion (BIC) stepwise selection in the discovery (left), validation (middle), and confirmation cohorts (right). (**E**) Time-dependent AUC of multivariate models over time in the discovery cohort. (**F**) Hazard ratio of plasma vRNA when sampled at DSO5, DSO9, or DSO13. HR adjusted for age and sex or not. Discovery: *n* = 61; validation: *n* = 87; confirmation: *n* = 69. For (F), all three cohorts were combined, complemented by 62 patients sampled before DSO7 (total *n* = 279).

As large studies have shown associations of older age and male sex with severe COVID-19 (*34*), we predefined adjustment by age and sex in the models. In the discovery cohort, time-dependent ROC for plasma vRNA showed a strong predictive capacity at DSO60 (AUC = 0.84; 95% CI: 0.72 to 0.96) and a slight benefit when adjusting for age and sex (AUC = 0.87; 95% CI: 0.76 to 0.99) (Fig. 4A). When applied to the validation cohort at DSO60, vRNA again had a good predictive capacity (AUC = 0.75; 95% CI: 0.59 to 0.92) and a benefit when adjusting for age and sex (AUC = 0.85; 95% CI: 0.65 to 1.00) (fig. S4B). Therefore, vRNA is a strong predictor of fatality, and adjusting for age and sex improves its predictive power.

Next, we compared the time-dependent ROC curves for inflammatory and tissue damage markers of the discovery cohort (Fig. 4B). Multivariate model selection retained only one analyte: Angiopoietin-2. To compare predictive accuracies at DSO60, we selected three additional analytes highly significant (*P* ≤ 0.001) in univariate Cox (Fig. 2G): CytoScore, CXCL13, and RAGE (Fig. 4B). Although no individual inflammatory cytokine was selected, the CytoScore had a high AUC (0.83; 95% CI: 0.71 to 0.95). Of the two markers of tissue insult, only Angiopoietin-2 (AUC = 0.86; 95% CI: 0.55 to 1.00) remained significant (RAGE: AUC = 0.80; 95% CI: 0.33 to 1.00). The chemokine CXCL13 (AUC = 0.82; 95% CI: 0.70 to 0.94) also had good predictive accuracy. All AUC values remained quite stable over time (fig. S5D). In the validation cohort, only CXCL13’s AUC remained high (AUC = 0.84; 95% CI: 0.66 to 0.98) and significantly discriminatory of mortality (*P* < 0.05) (fig. S4C) over time (fig. S5D). These observations confirm that certain markers of tissue insult and chemokine, as well as the overall cytokine levels, were associated with mortality risk.

For antibody measurements, we observed, within the discovery cohort, overlap of the time-dependent ROC of all three measurements significant in univariate Cox (ADCC: AUC = 0.74; 95% CI: 0.44 to 1.00; Spike Ig: AUC = 0.71; 95% CI: 0.25 to 1.00; RBD-specific IgG: AUC = 0.71; 95% CI: 0.44 to 0.97) at DSO60 (Fig. 4C). However, the predictive value of all three measurements began to drop around DSO30 (fig. S5E). We then applied the analysis to the validation cohort. As the cell-based ADCC assay requires significant infrastructure and technical expertise that may not be available in all clinical settings, we removed ADCC from the validation list of variables and substituted it by the technically simple RBD-specific IgG, given their strong correlation (Fig. 3C). The time-dependent ROC curves in the validation cohort for Spike Ig (AUC = 0.60; 95% CI: 0.15 to 1.00) and RBD-specific IgG titers (AUC = 0.59; 95% CI: 0.06 to 1.00) were nonsignificant and lower than in the discovery cohort. RBD-specific IgG titers displayed best predictive accuracy of mortality at DSO30 when adjusted for age and sex (RBD-specific IgG: 0.81; 95% CI: 0.50 to 1.00) (fig. S5G and table S2). Together, these data reveal that the anti–SARS-CoV-2 antibody response is highly associated with mortality within 30 days of symptom onset, but less so afterward.

After examining each variable in the setting of their category, we sought to identify which single parameter, or combination thereof, is the most robust. All variables selected by multivariate model within each category were considered for a global multivariate model, and age and sex covariates were forced regardless of their significance. In the discovery cohort, the variables selected in the global multivariate model (at DSO60: AUC = 0.91; 95% CI: 0.60 to 1.00) were vRNA (HR = 2.47; 95% CI: 1.30 to 4.68) and Angiopoietin-2 (HR = 4.22; 95% CI: 0.66 to 26.78), alongside the forced variables age (HR = 1.06; 95% CI: 0.99 to 1.10) and sex (HR = 0.94; 95% CI: 0.24 to 3.70) (Fig. 4D). Only vRNA (*P* = 0.006) remained independently associated with a higher risk of all-cause mortality within DSO60 in the global multivariate model. Of note, this global multivariate model was only slightly better than the three-variable model of vRNA, age, and sex at DSO60 (AUC = 0.87; 95% CI: 0.76 to 0.99) (Fig. 4D). In the validation cohort, the predictive accuracy of the model combining vRNA, Angiopoietin-2, age, and sex did not reach statistical significance (at DSO60: AUC = 0.86; 95% CI: 0.29 to 1.00) (Fig. 4D). However, the exclusion of Angiopoietin-2 improved the model’s discrimination in the validation cohort: the three-variable model combining vRNA, age, and sex was then significant (AUC = 0.85; 95% CI: 0.66 to 1.00). In both discovery and validation cohorts, the predictive accuracy of this model remained stable over time (Fig. 4E, fig. S5G, and table S2). We confirmed the predictive accuracy of plasma vRNA in a third cohort and again saw a significant association of the three-variable model with fatality (AUC = 0.90; 95% CI: 0.84 to 0.96) (table S2 and Fig. 4D).

A number of clinical scores and laboratory measurements have been developed for risk stratification of acutely ill patients. We therefore compared the predictive capacity of plasma vRNA with that of other measures taken in the clinical setting, namely, two metrics of organ failure: the quick sequential organ failure assessment (qSOFA) score (*35*) and the ratio of partial arterial oxygen pressure and fraction of inspired oxygen (P/F ratio) (*36*), as well as plasma concentrations of C-reactive protein (CRP) (*19*). All three variables were significantly associated to fatality in univariate analysis and when corrected for age and sex but inferior to plasma vRNA (table S2 and fig. S4, F and G). When combined in a multivariate with this latter parameter, qSOFA, P/F ratio, and CRP were no longer significant (table S2).

Last, we assessed the predictive accuracy of plasma vRNA when measured outside of the DSO11 time frame. We observed that as early as DSO5, plasma vRNA was already predictive of fatality and remained so at least until DSO13 (table S4 and Fig. 4F). This observation highlights the flexibility of using plasma vRNA for risk stratification, including at very early time points. Together, these data indicate that, at DSO11, measuring plasma SARS-CoV-2 vRNA in hospitalized patients with COVID-19 can be a powerful tool to predict mortality.

## DISCUSSION

In the perspective of clinical translation, it is essential to rigorously prioritize among the multitude of markers linked to COVID-19–related mortality. In patients with a spectrum of disease severity, we studied perturbations within three categories of plasma molecules: circulating SARS-CoV-2 vRNA (*14*), immune and tissue injury markers (*29*), and SARS-CoV-2–specific antibody responses (*26*), all of which can be probed by quick and technically robust assays. Strong associations of early parameters with the primary outcome, fatality within 60 days of symptom onset, were observed and largely maintained when the analyses were restricted to the critical group of patients on mechanical ventilation. Multivariate analyses demonstrated that, because of collinearity between several variables, a limited number of biological features was sufficient to build robust models predicting mortality. SARS-CoV-2 vRNA stood out as an early feature strongly associated with higher mortality risk. The predictive accuracy of plasma vRNA was superior to that observed with the clinical qSOFA and P/F ratio and the clinical CRP quantitation. Combined analysis of SARS-CoV-2 vRNA, Angiopoietin-2, age, and sex had greatest predictive accuracy in a discovery cohort, although a simpler model with vRNA, age, and sex was almost as robust. This three-parameter model maintained significant and very consistent predictive accuracy in a validation cohort and a confirmation cohort. Plasma vRNA remained predictive of fatality when sampled as early as DSO5 or as late as DSO13, indicating that it is an accurate predictor of fatality throughout the typical time of COVID-19–associated hospitalization (DSO7) and ICU admission (DSO10) (*17*, *37*).

The strength of the association between plasma vRNA levels and mortality risk was stronger than previously reported for NSW (*38*). In contrast to plasma, quantification of vRNA in NSW is hard to normalize, varies between types of tests, and depends on sample quality. Cox models showed a threefold increase in fatal outcome for every 1-U increase in log-transformed plasma vRNA quantity. While this association is reminiscent of the remarkable predictive value of plasma viral load for disease progression in untreated HIV-1 infection (*39*), no study has thus far convincingly demonstrated that therapeutic reduction of SARS-CoV-2 viral loads decreases mortality risk. For example, the antiviral remdesivir reduced viral loads in NSW, duration of symptoms, and hospitalization but had no significant impact on survival (*40*, *41*). Similarly, although monoclonal anti-Spike antibodies can reduce viral load (*42*, *43*), trials have not yet shown benefit in hospitalized patients. Given disease heterogeneity, it will be important to determine whether such interventions specifically benefit the subgroup of patients with high plasma vRNA.

The source and precise nature of the plasma vRNA remain to be better determined. Viral nucleic acids in the plasma do not prove the presence of replication-competent viral particles, as they could be viral debris translocated from damaged lung tissue. This is supported by the correlation we saw between vRNA and RAGE: As RAGE mRNA was not expressed in the peripheral blood mononuclear cells (PBMCs) of severe COVID-19 (*29*), plasma RAGE likely originates from damaged tissue (*31*). Besides the cytopathic effects of SARS-CoV-2 on lung epithelium, immunopathological mechanisms likely play key roles in severe COVID-19 pathogenesis (*44*). Systemic vRNA may trigger pathogen recognition receptors such as Toll-like receptors, in line with strong co–up-regulation of interferon-stimulated genes and other inflammatory pathways in vRNA-containing cells (*45*). This could contribute to the strong correlation observed between the amount of vRNA and IL-6, a pathogen-associated molecular pattern–triggered inflammatory cytokine (*46*).

Consistent with previous studies (*19*, *20*), we found significant associations between levels of several immune and tissue damage markers with both disease severity and mortality. Despite strongly significant HR for fatality risk for some analytes, the small sample size of our study resulted in sizable overlaps between CIs and variable rankings of HR values between the discovery and validation cohorts. An integrated CytoScore partially compensated for individual marker variability by giving an overall assessment of the magnitude of the cytokine storm. Notable individual markers were associated with fatal outcome, including Angiopoietin-2, CXCL13, and RAGE. While Angiopoietin-2 was less strongly correlated with vRNA than RAGE, it appears of significant interest in severe COVID-19 (*47*). This angiogenic factor has pro-inflammatory effects on the vascular endothelium, can disrupt vascular integrity and has been associated with ARDS (*48*), and might be a potential druggable target. We also observed a strong correlation of these markers of lung and vascular damage with plasma vRNA levels, which complement other reports showing a similarly strong association with biomarkers of heart and kidney damage (*49*).

Antibody responses likely contribute to viral control in acute SARS-CoV-2 infection (*16*, *26*), supported by the negative associations we observed between plasma vRNA and SARS-CoV-2–specific antibody responses. Whereas the antibody levels between the critical and noncritical groups were similar, mortality was overrepresented among patients who, at DSO11, had low RBD-specific IgG and low total Spike-binding Ig, although not in those with low RBD-specific IgM responses. Low IgG isoform among RBD-specific antibodies of deceased patients may indicate a disruption in B cell functions requiring T cell help, like class switching to IgG, possibly linked to inadequate T follicular helper (T_FH_) and/or germinal center (GC) disruption (*50*). CXCL13 is a key chemokine for recruitment to the GC of T_FH_ and B cells (*51*), and plasma CXCL13 is a marker of GC activity (*52*). The positive associations of CXCL13 levels with vRNA loads and fatality risk and the inverse correlation of CXCL13 levels with antibody responses may seem paradoxical, but high amounts of circulating CXCL13 might disrupt the dynamics of B cell recruitment to GCs. In addition, heightened systemic inflammation can impair development of adaptive immunity (*53*, *54*). These mechanisms may converge to reduce RBD-specific IgG responses in patients who succumb to their infection.

Defective early ADCC responses were also significantly associated with fatality, whereas we found only a nonsignificant trend for neutralization capacity. These observations support the idea that Fc-mediated functions could be important in controlling SARS-CoV-2, in line with recent reports showing that compromised Fc receptor binding strongly correlated with COVID-19 mortality (*26*), and Spike-specific humoral responses, including higher Fc effector functions, were enriched among survivors (*55*). Furthermore, antibodies with intact Fc effector functions were required for optimal protection against infection and correlated with decreased viral loads in animal models (*56*, *57*).

A limitation of our study is that we focused on inpatients who were usually hospitalized following worsening of their clinical condition, this occurring typically a few days after symptom onset. At this stage, some critical pathogenesis events have likely already occurred, which may narrow the window for some targeted interventions. This also excluded patients who were discharged early in their hospitalization. Complementary outpatient studies at very early time points will help identify factors that predict this initial worsening and determine their overlap with the features detailed here.

The significant interactions we observed between a number of the features measured are compatible with different, nonmutually exclusive mechanisms. Poor development of protective antibody responses may allow persistently high levels of viral replication, which, in turn, will lead to a cytokine storm. Conversely, high cytokine levels, perhaps driven by systemic vRNA, may disrupt adaptive immune responses. Although our observational study does not allow addressing the question of causation between the immunovirological alterations observed, these measurements can be useful tools to understand heterogeneity in disease trajectories and response to therapy, particularly in the context of large, well-controlled randomized trials. High viral loads and low levels of SARS-CoV-2–specific IgG may be mitigated through antivirals, monoclonal antibodies, or convalescent plasma therapy with high IgG content. People with high levels of selected cytokines may benefit the most from targeted immunotherapies. While recent trials have already resulted in improvement in clinical patient care, the predictive accuracy of plasma vRNA we observed and validated in patients hospitalized during the first COVID-19 wave was confirmed in patients recruited during the second and third waves. Still, it will be important to assess how new therapeutic strategies affect the potential of such immunovirological monitoring not only to predict outcome but also to individualize patient management.

## MATERIALS AND METHODS

### Participants and samples

SARS-CoV-2–positive patients admitted to the Centre Hospitalier de l’Université de Montréal (CHUM) or the Jewish General Hospital (JGH) were recruited into the Biobanque Québécoise de la COVID-19 (BQC19) (*58*). Samples from CHUM made up the discovery and confirmation cohort, and samples from JGH were the validation cohort. Blood draws were performed at baseline and, when possible, at day 2 (±3 days) and day 7 (±3 days) after enrollment. The study was approved by the respective institutional review boards and written informed consent was obtained from all participants or, when incapacitated, their legal guardian before enrollment and sample collection. Blood draws were also performed on 50 asymptomatic, NSW PCR-negative UC.

Hospitalized patients with COVID-19 were stratified on the basis of severity of respiratory support at the DSO11 time point: Critical patients required mechanical ventilation (endotracheal intubation, noninvasive ventilation, or ECMO); noncritical patients, encompassing both patients with moderate disease (required no supplemental oxygen) and patients with severe disease (required nasal cannula for oxygen). Mortality was followed up to 60 days. Medical charts were reviewed by two physicians for data collection on demographics, comorbidities, risk factors, severity state, time of infection, etc. (see Table 1). Median age and range for UC cohort were 37 (32 to 46), and 30 individuals were males (60%).

### Quantification of SARS-CoV-2 RNA

Absolute copy numbers of SARS-CoV-2 RNA (N region) in plasma samples were measured by real-time PCR. Total RNA was extracted from 230 μl of plasma collected on acid citrate dextrose using the QIAamp Viral RNA Mini Kit (Qiagen cat. no. 52906). Two master reaction mixes with specific primers and probes were prepared for quantification of N gene from SARS-CoV-2 and 18*S* (as a control for efficient extraction and amplification). Absolute copy numbers were measured by real-time PCR. Positive and no-template controls were included in all experiments. Purified RNA N transcripts (1328 base pairs) were quantified by NanoDrop, and the RNA copy numbers were calculated using the ENDMEMO online tool (see STAR methods for details).

### Measurements of plasma analytes by beads array

Duplicates of SARS-CoV-2–inactivated plasma samples were analyzed using a customized Human Magnetic Luminex Assay (LXSAHM-26, R&D; see table S1 for analyte list). Some cytokines and tissue damage markers were at very low concentrations, and the quantification platform we used was not sensitive enough to reliably quantify them in most samples. As such, analytes with extrapolated values for >90% of samples and/or negative values >15% of samples were identified by ∅ in Figs. 2 and 3 and fig. S2.

### CytoScore

For *k* analytes (*n* = 26), the CytoScore for each sample was calculated as follows

$$\frac{\sum_{n=1}^{k}\frac{c_{n}-\mu_{n}^{\text{UC}}}{\sigma_{n}^{\text{UC}}}}{k}$$

where *c_n_* is the concentration for analyte *n*, $\mu_{n}^{\text{UC}}$ is the mean concentration of UC samples for analyte *n*, and $\sigma_{n}^{\text{UC}}$ is the SD of UC samples for analyte *n*.

### Serology measurements

Plasma from uninfected donors were used as negative controls and used to calculate the seropositivity threshold in our ELISA and flow cytometry assays. The monoclonal antibody CR3022 (*59*) was used as a positive control.

### RBD-specific ELISA

The SARS-CoV-2 RBD ELISA used was recently described (*24*). The seropositivity threshold was established using the following formula: mean of all COVID-19–negative plasma + (3 SD of the mean of all COVID-19–negative plasma) (see the Supplementary Materials for details).

### Flow cytometry analysis of cell surface staining

As recently described (*24*), plasma from SARS-CoV-2–infected or uninfected individuals (1/250 dilution) was added onto 239T cells expressing Spike and green fluorescent protein (GFP). Alexa Fluor 647–conjugated goat anti-human IgG (H+L) antibodies (Invitrogen) were used as secondary antibodies. The seropositivity threshold was established using the following formula: mean of all COVID-19–negative plasma + (3 SD of the mean of all COVID-19–negative plasma + inter-assay coefficient of variability) (see the Supplementary Materials for details).

### Virus neutralization assay

As recently described (*24*), 293T-ACE2 target cells were infected with single-round luciferase-expressing pseudoparticles bearing the SARS-CoV-2 Spike in the presence of patient plasma at different dilutions. The neutralization half-maximal inhibitory dilution (ID_50_) represents the plasma dilution to inhibit 50% of the infection of target cells (see the Supplementary Materials for details).

### ADCC assay with SARS-CoV-2 Spike-expressing cells

As previously described (*60*), patient plasma was tested for ADCC activity against CEM-NKr cells stably expressing the full length GFP-tagged SARS-CoV-2 Spike (CEM.NKr. Spike^+^) and effector cells (stained PBMCs) were mixed at a ratio of 1:10 (*61*). Plasma from COVID-19 infected or uninfected individuals (1/500 dilution) was added, and cocultures were incubated for 6 hours. ADCC was calculated by gating on Spike-expressing live target cells and using the formula

$$\%\text{ADCC}=\frac{\%{\text{GFP}}_{\text{targets}+\text{effectors}}-\%{\text{GFP}}_{\text{targets}+\text{effectors}+\text{plasma}}}{\%{\text{GFP}}_{\text{targets}}}\times 100$$

%ADCC obtained with plasma was further normalized to positive control. The specificity threshold was established using the following formula: mean of all COVID-19–negative plasma + 3 SD of the mean of all COVID-19–negative plasma.

#### Clinical scores

The qSOFA and P/F ratios were calculated on the basis of data clinically collected into the patients’ medical record of the hospital stay. The qSOFA score was calculated as previously described (*35*). This three-point score assigns one point for low blood pressure (systolic blood pressure ≤ 100 mmHg), high respiratory rate (≥22 breaths per minute), or altered mentation. The ratio of partial arterial oxygen pressure and fraction of inspired oxygen (P/F ratio) was approximated on the basis of the oxygen saturation measured by pulse oximetry and the fraction of inspired oxygen by nonlinear imputation, as previously described (*36*).

#### CRP quantitation

The measurement of CRP in plasma was performed by the clinical biochemistry laboratories of the respective hospitals where patients were recruited (CHUM and JGH).

### Statistical analyses and multivariate models

#### Methods for univariate models

The association between measured variables and time to death was analyzed by Cox proportional survival hazard. The dependent variable in all survival analyses was time to death during the follow-up, measured in days. Subjects were censored upon reaching 60 days of follow-up (no patients withdrew within this time frame). Time 0 was defined as the day of symptom onset. Univariate Cox proportional hazard regression was used to determine the association between plasma analytes and all-cause mortality at DSO60 for all COVID-19–positive patients, as well as critical patients’ subgroup only. Analytes were log-transformed when they naturally followed exponential distribution, for example, vRNA and cytokines. Next, the estimated survival proportions at any given point in time for undetectable (when applicable), low (lower interquartile range level of detectable), or high (upper interquartile range of detectable) amounts of analyte considered (plasma vRNA, CytoScore or antibody responses) were extracted from Cox models (*28*) and presented in the graphical form (*28*).

#### Part 1: Multivariate Cox model

Potential risk factors were grouped into three categories: (i) vRNA, (ii) 26 cytokines and tissue damage variables, and (iii) six antibody-associated variables. Model building was performed in three steps. In the first step, univariate models for risk factor of death by DSO60 were developed, one for each of the covariates in the category; only risk factors with *P* value <0.05 were retained. For the second category of 26 cytokines, an additional criterion of variable selection was applied to ensure the quality of the measurements: the cytokines or tissue damage markers with extrapolated values for >90% of samples and/or negative values >15% of samples were excluded for future investigation. These exclusion criteria were added as the quantification platform we used was not sensitive enough to reliably quantify some low-concentration analytes, and we wanted to rely on analytes that are well quantified for our multivariate model. Of 26 cytokines and tissue-damage markers, 19 satisfied these criteria. In the second step, we focused on categories for which more than one variable had been retained in the first step; then, the stepwise Cox model selection based on the Bayesian information criterion (BIC) was used to obtain the most parsimonious model (lowest value) for each of these categories. This penalized likelihood criterion selects the best variable at predicting data and then adds one additional variable at a time while accounting for potential overfitting, in the end only selecting the multivariate model with the lowest BIC value, i.e., the most parsimonious. In addition, to keep the risk of overfitting low, no more than six predictor parameters were entered in the multivariate model for our sample of 61 patients (*62*, *63*).

In the third step, all variables retained in the second step were considered; then, the BIC was used to obtain a global parsimonious model. On the basis of the literature (*64*), age and sex are associated with the mortality for patients with COVID-19; however, in the small homogeneous sample, it might be hard to detect these relations. Thus, in each model, age and sex covariates were forced in the multivariate model regardless of their significance. Potential interactions between each covariate with age and sex were tested to verify whether the effect was consistent across different age and between sex. Potential presence of multicollinearity was assessed by calculating the variance inflation factor for each variable. This allowed us to identify and treat in separate models subsets of covariates, which were highly correlated.

#### Part 2: Time-dependent ROC curve

To evaluate predictive accuracy of survival models, the time-dependent ROC curves for right-censored data (*65*) were calculated, compared across different Cox models, and presented in the graphical form. The inverse probability of censoring weighting technique was used for estimating time-dependent ROC curves (*66*). The AUC was examined at 60 days as well at all distinct event times by plotting the AUC and the 95% confidence limits over time. Day 48 corresponds to the last event (fatality) day in the discovery cohort.

#### Part 3: Independent cohort validation

All final multivariate Cox models were reassessed in the validation and confirmation cohorts by independently executing the multivariate models with the same list of variables obtained, in the discovery cohort, in steps 2 and 3. Then, using the same approach described above, the time-dependent ROC curves were evaluated in the validation dataset to validate our finding.

#### Sensitivity analysis

Two additional sensitivity analyses were performed. First, to compare the predictive capacity of the final selected model versus models with easily available clinical measures (qSOFA, P/F ratio, and CRP), the univariate and multivariate Cox regressions were presented. Discovery cohort was used for qSOFA and P/F ratio. Three study cohorts were combined for the analysis with CRP owing to partially available data in each cohort.

Second, to see how the final results were affected by earlier time point measurements and shorter time windows, three new datasets were extracted from longitudinal measurements of the combined cohorts at DSO5 (DSO3 to DSO7), DSO9 (DSO8 to DSO11), and DSO13 (DSO12 to DSO15) time frames. The final Cox regressions models were repeated for each dataset.

## Notes

View/request a protocol for this paper from *Bio-protocol*.
