# Supplementary material for: Integrated immunovirological profiling validates plasma SARS-CoV-2 RNA as an early predictor of COVID-19 mortality
Source: Sci Adv. 2021 Nov 26;7(48):eabj5629. doi: 10.1126/sciadv.abj5629 (PMC8626074; doi:10.1126/sciadv.abj5629)
Supplement: Supplementary file 1 — Figs. S1 to S5 Tables S1 to S4 [file sciadv.abj5629_sm.pdf]

Science Advances
AAAS

Supplementary Materials for

Integrated immunovirological profiling validates plasma SARS-CoV-2 RNA as an early predictor of COVID-19 mortality

Elsa Brunet-Ratnasingham, Sai Priya Anand, Pierre Gantner, Alina Dyachenko, Gaël Moquin-Beaudry, Nathalie Brassard, Guillaume Beaudoin-Bussières, Amélie Pagliuzza, Romain Gasser, Mehdi Benlarbi, Floriane Point, Jérémie Prévost, Annemarie Laumaea, Julia Niessl, Manon Nayrac, Gérémy Sannier, Catherine Orban, Marc Messier-Peet, Guillaume Butler-Laporte, David R. Morrison, Sirui Zhou, Tomoko Nakanishi, Marianne Boutin, Jade Descôteaux-Dinelle, Gabrielle Gendron-Lepage, Guillaume Goyette, Catherine Bourassa, Halima Medjahed, Laetitia Laurent, Rose-Marie Rébillard, Jonathan Richard, Mathieu Dubé, Rémi Fromentin, Nathalie Arbour, Alexandre Prat, Catherine Larochelle, Madeleine Durand, J. Brent Richards, Michaël Chassé, Martine Tétreault, Nicolas Chomont*, Andrés Finzi*, Daniel E. Kaufmann*

*Corresponding author. Email: nicolas.chomont@umontreal.ca (N.C.); andres.finzi@umontreal.ca (A.F.); daniel.kaufmann@umontreal.ca (D.E.K.)

Published 26 November 2021, *Sci. Adv.* **7**, eabj5629 (2021)
DOI: 10.1126/sciadv.abj5629

This PDF file includes:

Figs. S1 to S5
Tables S1 to S4

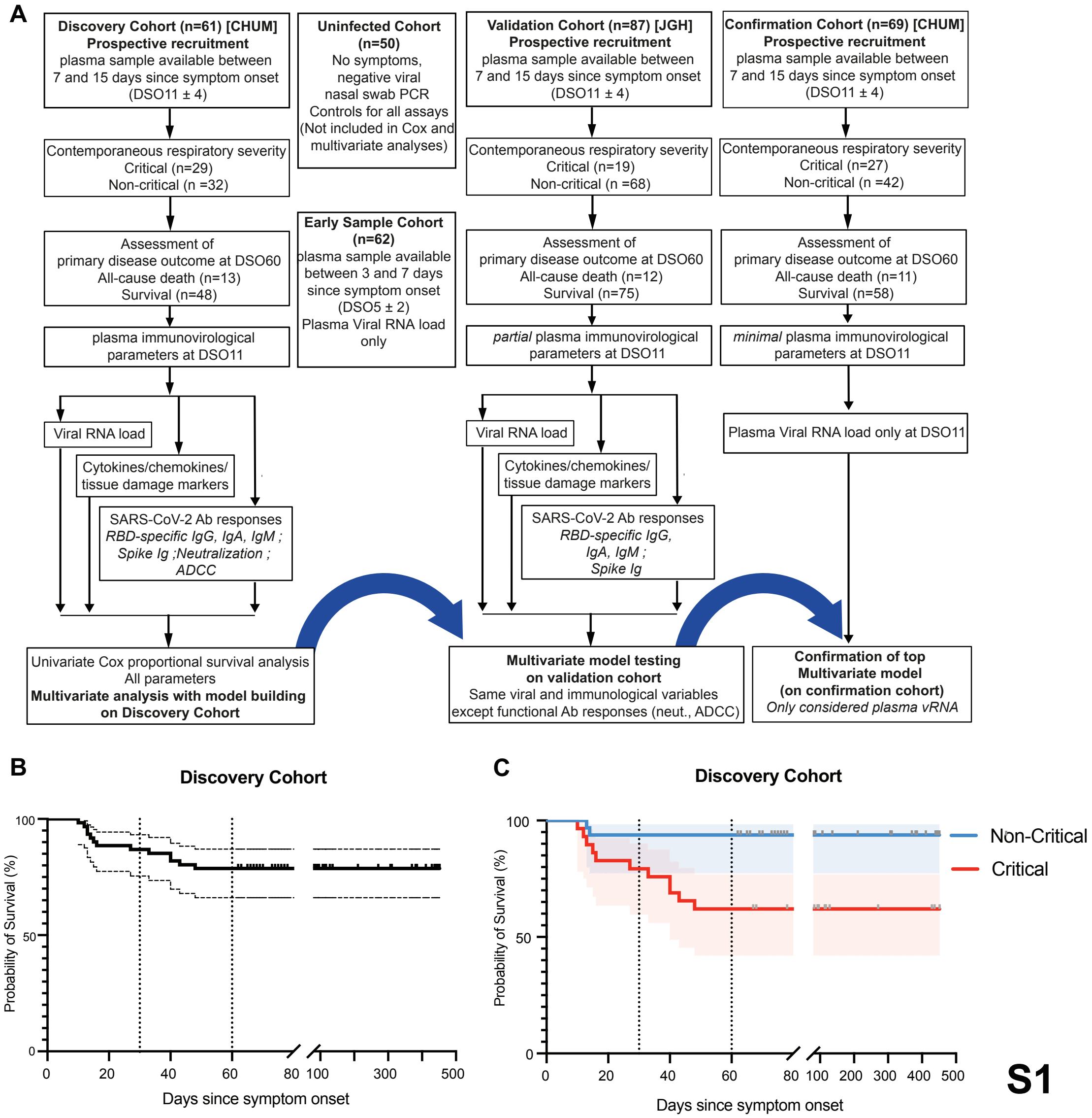

Fig. S1. Study design (A) Study Design and analyses performed on the three COVID-19 cohorts (discovery, n=61; validation, n=87; and confirmation, n=69) and the uninfected cohort. In addition, 62 patients who were sampled at very early time points (before DSO7) were included for longitudinal testing of selected models. (**B**) Survival curve in the discovery cohort based on days since symptom onset. (**C**) Within the discovery cohort, Kaplan-Meier analysis of survival in Non-critical (blue) compared to Critical (red) subgroups, whose disease severity was assessed at DSO11. Curves compared using Log-rank (Mantel-Cox) test. n = 61 COVID-19 patients (13 fatalities).

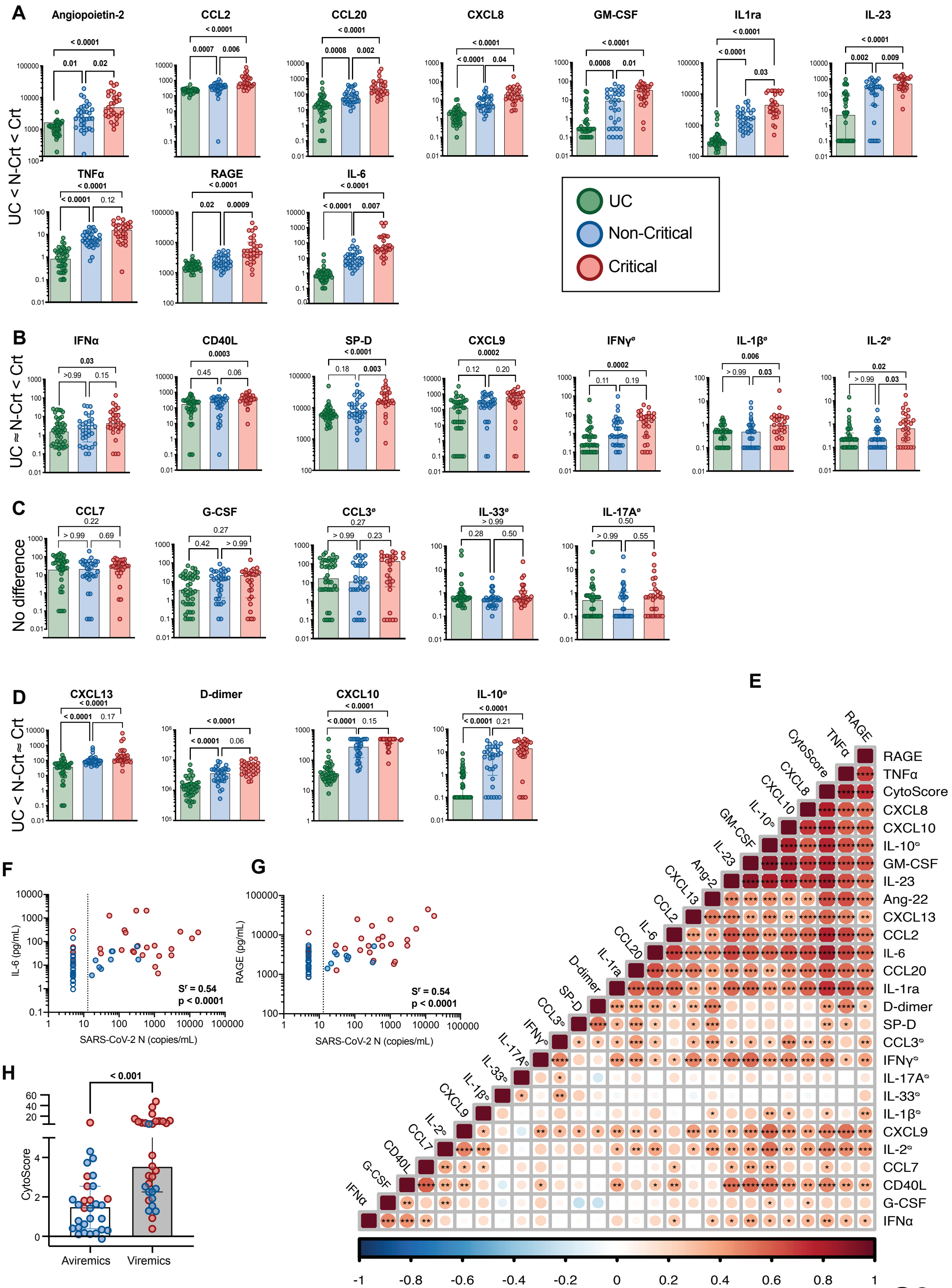
A
Angiopoietin-2
CCL2
CCL20
CXCL8
GM-CSF
IL1ra
IL-23
TNFα
RAGE
IL-6
UC < N-Crt < Crt
UC
Non-Critical
Critical
B
IFNα
CD40L
SP-D
CXCL9
IFNγ
IL-1β
IL-2
UC ≈ N-Crt < Crt
C
CCL7
G-CSF
CCL3
IL-33
IL-17A
No difference
D
CXCL13
D-dimer
CXCL10
IL-10
UC < N-Crt ≈ Crt
E
RAGE
TNFα
CytoScore
CXCL8
CXCL10
IL-10
GM-CSF
IL-23
Ang-22
CXCL13
CCL2
IL-6
CCL20
IL-1ra
D-dimer
SP-D
CCL3
IFNγ
IL-17A
IL-33
IL-1β
CXCL9
IL-2
CCL7
CD40L
G-CSF
IFNα
F
IL-6 (pg/mL)
SARS-CoV-2 N (copies/mL)
Sr = 0.54
p < 0.0001
G
RAGE (pg/mL)
SARS-CoV-2 N (copies/mL)
Sr = 0.54
p < 0.0001
H
< 0.001
CytoScore
Avirémics
Virémics

S2

Fig. S2. Inflammatory cytokines, chemokines and markers of tissue damage are increased in critical cases of COVID-19. (A-D) Comparison of cytokine concentrations between critical COVID-19, non-critical COVID-19 and UC. Cytokines and markers of tissue damage grouped according to differential detection: (**A)** Greatest in critical (Crt), but also higher in non-critical (N-Crt) compared to UC; (**B)** Similar between UC and non-critical, but greater in critical COVID-19; (**C)** No differences between all three groups; (**D)** Greater in COVID-19 compared to UC, but similar between non-critical and critical. (**E)** Correlation matrix of all 26 plasma analytes and CytoScore (see methods for details on CytoScore). Color of circle represents Spearman R value (red = 1, blue = -1) and respective p values are represented by * within circles ($p < 0.05$ = *; $p < 0.01$ =** ; $p<0.001$=*** ; $p<0.0001$=****). (**FG)** Correlation of plasma vRNA and plasma concentration of (**F)** IL-6 or (**G)** RAGE (pg/mL). (**H)** Comparison of CytoScore between aviremics (<13 vRNA copies/mL) and viremics (≥ 13 copies/mL). Mann-Whitney test. A-D) Kruskall-Wallis with Dunn's multiple comparisons test. For A-D, F-H, color-coded dots represent severity of the patient at DSO11 (red = critical, blue = non-critical, green=UC). For A-E, cytokines with titles annotated by ∅ are poorly detected (see methods for details). n = 61 COVID-19 subjects (13 mortalities) and 43 UC.

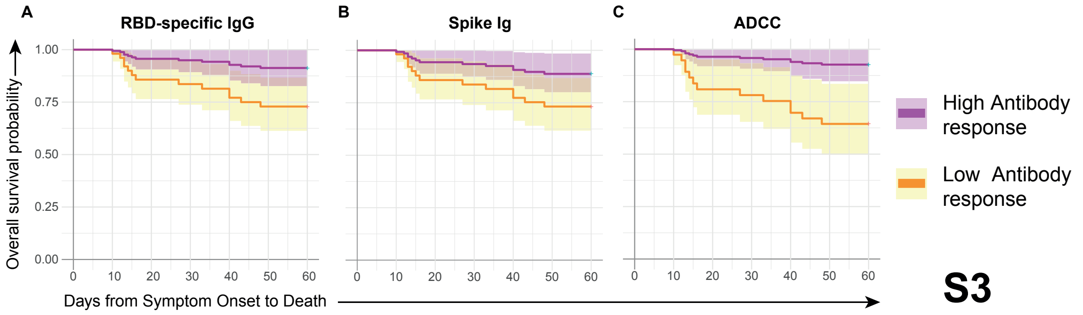

Fig. S3. Association of poor outcome with low RBD-specific IgG titers is maintained in the critical COVID-19 group. (A-C) Modelisation of the predicted survival curves of patients with high (orange) or low (purple) (**A)** RBD-specific IgG, (**B)** Spike-specific Ig or (**C)** ADCC activity in critical COVID-19 patients. n = 29 Critical COVID-19 cases (11 mortalities). The predicted values were calculated from the respective Cox regression models.

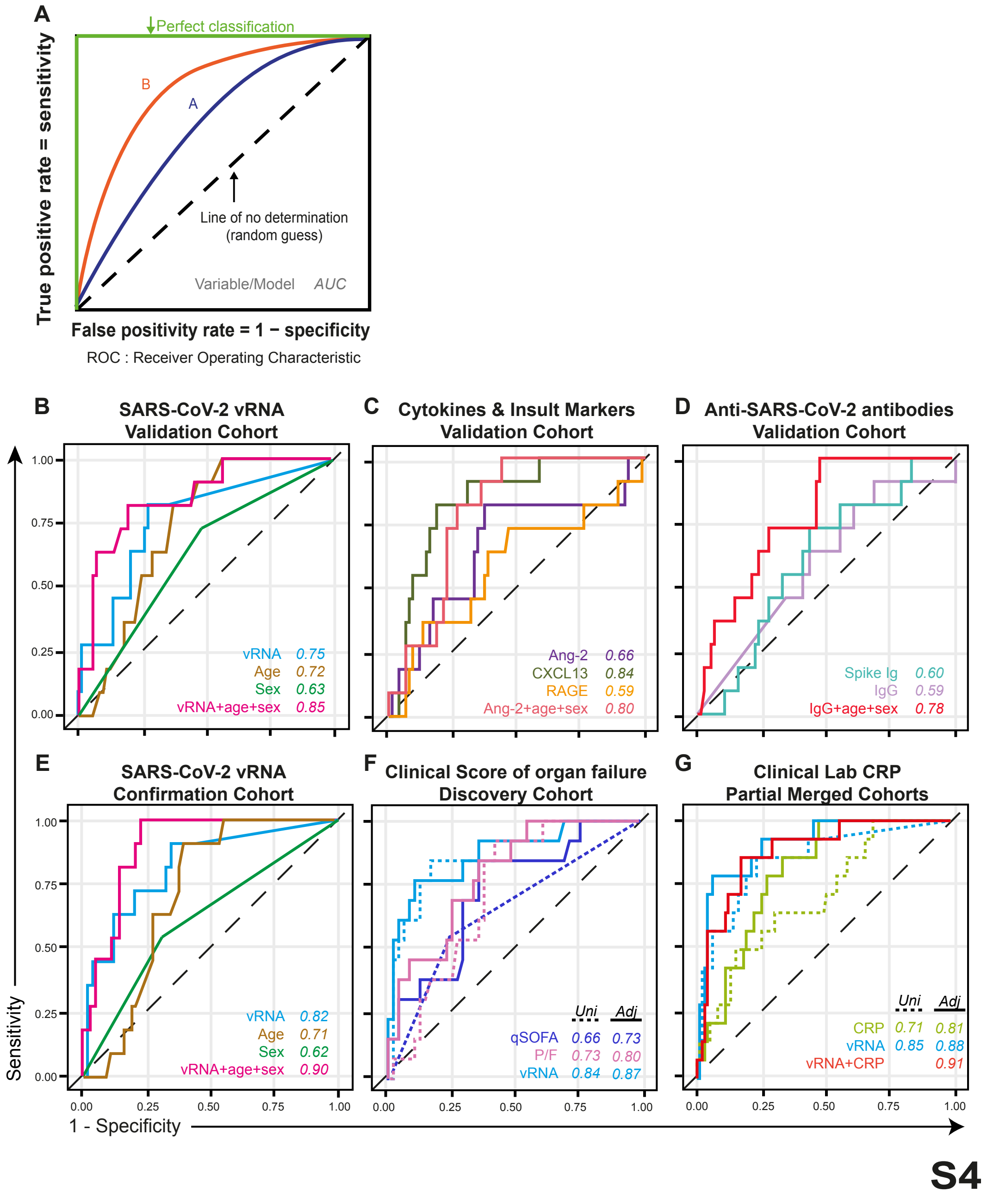

Fig. S4. Reproducibility of predictive accuracy for mortality in the validation and confirmation cohorts. **A**) Concept of time-dependent ROC curves. A ROC is defined by the false-positive rate and true-positive rate, which depicts relative trade-offs resulting from changing the test threshold. The best possible prediction model (100% sensitivity and 100% specificity) would yield a "square curve", reaching the upper left corner (green line). A completely random guess (chance) would give a point along the diagonal dotted black line (line of no determination). In the

present study, the ROC curves were used compare the predictive accuracy of different immunovirological parameters measured in plasma at DSO11. These ROC curves were time-dependent, meaning they vary depending on the time between symptom onset and death considered. Here, model B is superior to A. ROC curves are further characterized by AUC, a measure of test accuracy (1.0= best possible test; 0.5=no discrimination). **(B-G)** Time-dependent ROC curves measured (**B-D**) within the validation cohort for (**B)** plasma vRNA, age and sex, (**C)** Cytokines and tissue insult markers or (**D)** anti-SARS-CoV-2 antibody responses; (**E**) within the confirmation cohort for plasma vRNA, age and sex; (**F)** within the discovery cohort for quick SOFA (qSOFA) and P/F ratio; **(G)** within a combination of samples across cohorts (those for whom CRP clinical lab quantitation was available within the DSO11 time point) for CRP clinical lab measurements and vRNA in the same subset of patients. (B-G) Legends with color-coded variables are on the bottom left of panels, and values in italic are the AUC at 60 days after symptom onset associated to the variable or model. For (FG), ROC curves of adjusted values (age+sex) are in continuous lines, whereas models for univariate analyses are in dashed lines. AUC values are given at 30 days or 60 days after symptom onset. (B-D): n = 87; (E) n = 69; (F) n = 61; (G) n = 113. See Supplemental Table 2 for details.

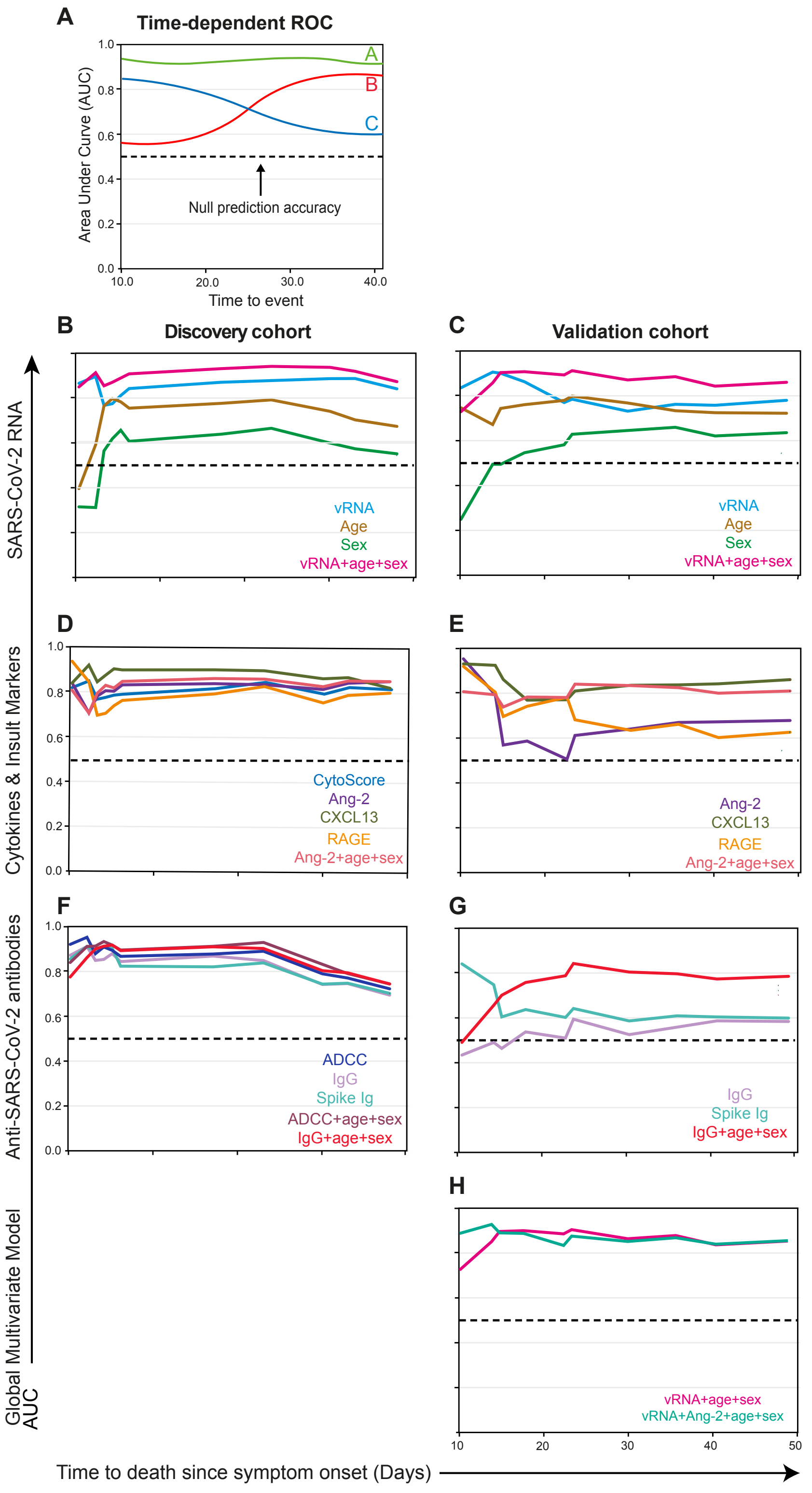

A
Time-dependent ROC
1.0
0.8
0.6
0.4
0.2
0.0
Area Under Curve (AUC)
A
B
C
Null prediction accuracy
10.0
20.0
30.0
40.0
Time to event
B
Discovery cohort
C
Validation cohort
SARS-CoV-2 RNA
vRNA
Age
Sex
vRNA+age+sex
D
E
Cytokines & Insult Markers
CytoScore
Ang-2
CXCL13
RAGE
Ang-2+age+sex
F
G
Anti-SARS-CoV-2 antibodies
ADCC
IgG
Spike Ig
ADCC+age+sex
IgG+age+sex
H
Global Multivariate Model
AUC
vRNA+age+sex
vRNA+Ang-2+age+sex
10
20
30
40
50
Time to death since symptom onset (Days)

S5

Fig. S5. Predictive accuracy of immunovirological markers over time, in the discovery and validation cohorts. **A**) Concept of time-dependent AUC changes. To observe changing accuracy overtime, AUCs for a given measurement were plotted against time to death. AUC values closer to 1 (top) have better predictive accuracy; AUC values close to 0.5 have poor prediction accuracy. In this example, measurement A maintained the greatest prediction accuracy throughout time, whereas C > B before DSO30, then B > C after DSO30. (**B)** Time-dependent AUC of plasma vRNA, age and sex for validation cohort; (**CD)** Time-dependent AUC of plasma cytokines and tissue damage markers for (**C**) discovery cohort or (**D**) validation cohort; **(EF)** Time-dependent AUC of SARS-CoV-2 antibody responses for (**E**) discovery cohort or (**F**) validation cohort. (**GH**) Time-dependent AUC for top measurements captured by multivariate model analysis in the (**G**) discovery cohort or (**H**) validation cohort. n = 61 for discovery cohort; 87 for validation cohort. Legends with color-coded variables are on the bottom left of panels, and values in italic are the AUC values associated to the variable.

Table S1. Full list of analytes measured in plasma by beads arrays

Analyte	Bead Region
Angiopoietin-2	26
CCL3/MIP-1 alpha	35
CCL20/MIP-3 alpha	33
CXCL9/MIG	52
CXCL13/BCA-1	28
G-CSF	54
IFNα	63
IL-1β/IL-1F2	57
IL-2	27
IL-8/CXCL8	18
IL-17/IL-17A	43
IL-33	14
SP-D	62
CCL2/JE/MCP-1	25
CCL7/MCP-3/MARC	37
CD40 Ligand/TNFSF5	74
CXCL10/IP-10/CGR-2	21
D-dimer	43
GM-CSF	46
IFN-γ	29
IL-1ra/IL-1F3	30
IL-6	13
IL-10	22
IL-23	76
RAGE/AGER	45
TNFα	12

Human Magnetic Luminex ® Assays, from R&D Systems (Biotechne)
Premixed Multiplex
Kit Catalog Numbers : LXSAHM-26
Kit Lot Number : L134818

Table S2. Time-dependent AUC for representative variables per category in all three cohorts.

Cohort	Model[a]		DSO30[b]	DSO60[b]	Overall: IAUC[c]	Maximum AUC[d]	Time Max AUC[e]
Discovery n=61	vRNA	Not-adjusted	**0.87 (0.76, 0.98)**	**0.84 (0.72, 0.96)**	0.84	**0.90 (0.83, 0.96)**	12
		Adjusted	**0.93 (0.82, 1.00)**	**0.87 (0.76, 0.99)**	0.90	**0.94 (0.84, 1.00)**	33
	Ang-2	Not-adjusted	**0.85 (0.53, 1.00)**	**0.86 (0.55, 1.00)**	0.82	**0.86 (0.55, 1.00)**	48
		Adjusted	**0.87 (0.54, 1.00)**	**0.86 (0.54, 1.00)**	0.83	**0.87 (0.54, 1.00)**	27
	IgG	Not-adjusted	**0.88 (0.60, 1.00)**	0.71 (0.44, 0.97)	0.84	**0.92 (0.63, 1.00)**	12
		Adjusted	**0.93 (0.71, 1.00)**	**0.76 (0.58, 0.95)**	0.88	**0.94 (0.71, 1.00)**	15
	vRNA + Ang-2	Not-adjusted	**0.91 (0.58, 1.00)**	**0.91 (0.60, 1.00)**	0.88	**0.93 (0.60, 1.00)**	43
		Adjusted	**0.95 (0.64, 1.00)**	**0.91 (0.62, 1.00)**	0.91	**0.96 (0.65, 1.00)**	33
Validation n=87	vRNA	Not-adjusted	**0.73 (0.55, 0.91)**	**0.75 (0.59, 0.92)**	0.82	**0.92 (0.82, 1.00)**	12
		Adjusted	**0.89 (0.67, 1.00)**	**0.85 (0.65, 1.00)**	0.89	**0.93 (0.70, 1.00)**	22
	Ang-2	Not-adjusted	0.63 (0.13, 1.00)	0.66 (0.14, 1.00)	0.65	0.95 (0.19, 1.00)	8
		Adjusted	0.83 (0.28, 1.00)	0.80 (0.26, 1.00)	0.79	0.84 (0.29, 1.00)	22
	IgG	Not-adjusted	0.52 (0.04, 1.00)	0.59 (0.06, 1.00)	0.53	0.59 (0.08, 1.00)	22
		Adjusted	0.81 (0.50, 1.00)	0.78 (0.48, 1.00)	0.75	**0.85 (0.52, 1.00)**	22
	vRNA + Ang-2	Not-adjusted	0.74 (0.24, 1.00)	0.77 (0.25, 1.00)	0.81	0.96 (0.30, 1.00)	12
		Adjusted	0.87 (0.30, 1.00)	0.86 (0.29, 1.00)	0.90	0.95 (0.32, 1.00)	12
Confirmation n=69	vRNA	Not-adjusted	**0.77 (0.53, 1.00)**	**0.82 (0.68, 0.97)**	0.83	**0.98 (0.95, 1.00)**	19
		Adjusted	**0.90 (0.81, 1.00)**	**0.90 (0.84, 0.96)**	0.91	**0.95 (0.85, 1.00)**	19

Values are AUC (95%CI), and AUC at $p<0.05$ are in bold.

[a] AUC values given either not adjusted (only variable(s) listed) or adjusted (age and sex).

[b] AUC values are given at 30 days or 60 days after symptom onset

[c] Integrated AUC (IAUC) is the average of all AUC from DSO10-60.

[d] Maximum AUC is the best prediction accuracy of the variable (measured at DSO11)

[e] Date since symptom onset at which that maximum AUC was achieved

Table S3. Comparison between time-dependent AUC for clinically-collected variables and plasma vRNA.

Cohort	Variable[a]		HR (95%CI)	P value	AUC at DSO60[b]	IAUC[c]
Discovery (n=61)	vRNA[d]	Not Adjusted	**3.13 (1.90, 5.17)**	**< 0.001**	**0.84 (0.72, 0.96)**	**0.84**
		Adjusted	**3.53 (2.03, 6.16)**	**< 0.001**	**0.87 (0.76, 0.99)**	**0.90**
	qSOFA[e]	Not Adjusted	**3.25 (1.09, 9.70)**	**0.03**	0.65 (0.43, 0.88)	0.66
		Adjusted	**3.35 (1.10, 10.23)**	**0.03**	**0.73 (0.58, 0.88)**	**0.76**
	P/F[f]	Not Adjusted	**0.91 (0.85, 0.98)**	**0.02**	**0.73 (0.59, 0.86)**	**0.78**
		Adjusted	**0.91 (0.84, 0.98)**	**0.01**	**0.80 (0.68, 0.91)**	**0.84**
	vRNA + qSOFA	Adjusted				
		vRNA	**3.65 (1.79; 7.44)**	**<0.001**	**0.85 (0.73; 0.97)**	**0.85**
		qSOFA	0.61 (0.13; 2.87)	0.53		
	vRNA + P/F	Adjusted				
		vRNA	**2.57 (1.46; 4.50)**	**0.001**	**0.88 (0.75; 1.00)**	**0.87**
		P/F	0.99 (0.99; 1.003)	0.24		
Merged Cohort[g] (n=113, 98 survivor and 15 non-survivor)	vRNA	Not Adjusted	**2.59 (1.78; 3.76)**	**< 0.001**	**0.85 (0.74; 0.96)**	**0.87**
	CRP[d]	Not Adjusted	**4.73 (1.44 ; 15.50)**	**0.01**	**0.71 (0.49; 0.93)**	**0.62**
	vRNA + CRP	Not Adjusted				
		vRNA	**2.69 (1.79; 4.05)**	**< 0.001**	**0.87 (0.75; 0.99)**	**0.86**
		CRP[d]	**4.15 (1.17; 14.68)**	**0.03**		
	vRNA + CRP	Adjusted				
		vRNA	**2.82 (1.72; 4.62)**	**< 0.001**	**0.91 (0.83; 0.99)**	**0.93**
		CRP[d]	**4.01 (0.97; 16.67)**	**0.06**		
		age	**1.05 (1.01; 1.09)**	**0.008**		
		sex	0.39 (0.10; 1.53)	0.18		

Values are Hazard ratio (HR) (95%Confidence interval CI) with p value, and AUC. p<0.05 are in bold.

[a] AUC values given either not adjusted (only variable(s) listed) or adjusted (age and sex). For multivariate models, AUC is given for each parameter *within* the model.

[b] AUC values are given at 60 days after symptom onset

[c] Integrated AUC (IAUC) is the average of all AUC from DSO10-60.

[d] HR value is given for every increase in 1 log unit.

[e] HR value is given for high qSOFA (>=2) vs low qSOFA (<=1) (categorical values).

[f] HR values given for every 10 unit increase.

[g] Includes patients among the three merged cohorts for whom clinical lab CRP measurements were available at DSO11.

Table S4. Predictive accuracy of plasma vRNA measured at different times after symptom onset.

Median DSO [range]	Total[#]	Non-survivor	Model[a]	Hazard Ratio		AUC	
				HR(95%CI)	P-value	AUC at DSO60[b]	IAUC[c]
DSO5 [3-7]	80	15	Not-adjusted	**2.32 (1.52, 3.55)**	**0.0001**	**0.76 (0.63, 0.89)**	**0.82**
			Adjusted	**2.03 (1.34, 3.06)**	**0.0007**	**0.83 (0.70, 0.96)**	**0.89**
DSO9 [8-11]	164	21	Not-adjusted	**3.03 (2.11, 4.34)**	**< 0.0001**	**0.86 (0.77, 0.95)**	**0.87**
			Adjusted	**2.82 (1.99, 4.00)**	**< 0.0001**	**0.89 (0.82, 0.97)**	**0.90**
DSO13 [12-15]	127	17	Not-adjusted	**2.88 (1.80, 4.62)**	**< 0.0001**	**0.77 (0.64, 0.90)**	**0.81**
			Adjusted	**3.37 (2.05, 5.54)**	**< 0.0001**	**0.82 (0.68, 0.95)**	**0.89**

[#] Total of 371 samples collected on 279 patients
Values are Hazard ratio (HR) (95%Confidence interval CI) with p value, and AUC. $p<0.05$ are in bold. All values relate to plasma vRNA (for an increase of 1 unit of copies/mL)
[a] HR and AUC values given either not adjusted (only variable(s) listed) or adjusted (age and sex).
[b] AUC values are given at 60 days after symptom onset
[c] Integrated AUC (IAUC) is the average of all AUC from DSO10-60.
